# Construction of an environmental quality index for public health research

**DOI:** 10.1186/1476-069X-13-39

**Published:** 2014-05-22

**Authors:** Lynne C Messer, Jyotsna S Jagai, Kristen M Rappazzo, Danelle T Lobdell

**Affiliations:** 1 School of Community Health; College of Urban and Public Affairs, Portland State University, Portland, OR, USA; 2 National Health and Environmental Effects Research Laboratory, U.S. Environmental Protection Agency, Chapel Hill, NC, USA; 3 School of Public Health, Division of Environmental and Occupational Health Sciences, University of Illinois, Chicago, Chicago, IL, USA; 4 Gillings School of Global Public Health, University of North Carolina, Chapel Hill, NC, USA; 5 Oak Ridge Institute for Science and Education, National Center for Environmental Assessment, U.S. Environmental Protection Agency, Research Triangle Park, Oak Ridge, NC, USA

**Keywords:** Environmental quality, Air quality, Water quality, Land quality, Built environment, Sociodemographic, Rural–urban status

## Abstract

**Background:**

A more comprehensive estimate of environmental quality would improve our understanding of the relationship between environmental conditions and human health. An environmental quality index (EQI) for all counties in the U.S. was developed.

**Methods:**

The EQI was developed in four parts: domain identification; data source acquisition; variable construction; and data reduction. Five environmental domains (air, water, land, built and sociodemographic) were recognized. Within each domain, data sources were identified; each was temporally (years 2000–2005) and geographically (county) restricted. Variables were constructed for each domain and assessed for missingness, collinearity, and normality. Domain-specific data reduction was accomplished using principal components analysis (PCA), resulting in domain-specific indices. Domain-specific indices were then combined into an overall EQI using PCA. In each PCA procedure, the first principal component was retained. Both domain-specific indices and overall EQI were stratified by four rural–urban continuum codes (RUCC). Higher values for each index were set to correspond to areas with poorer environmental quality.

**Results:**

Concentrations of included variables differed across rural–urban strata, as did within-domain variable loadings, and domain index loadings for the EQI. In general, higher values of the air and sociodemographic indices were found in the more metropolitan areas and the most thinly populated areas have the lowest values of each of the domain indices. The less-urbanized counties (RUCC 3) demonstrated the greatest heterogeneity and range of EQI scores (−4.76, 3.57) while the thinly populated strata (RUCC 4) contained counties with the most positive scores (EQI score ranges from −5.86, 2.52).

**Conclusion:**

The EQI holds promise for improving our characterization of the overall environment for public health. The EQI describes the non-residential ambient county-level conditions to which residents are exposed and domain-specific EQI loadings indicate which of the environmental domains account for the largest portion of the variability in the EQI environment. The EQI was constructed for all counties in the United States, incorporating a variety of data to provide a broad picture of environmental conditions. We undertook a reproducible approach that primarily utilized publically-available data sources.

## Background

Polluted environments have contributed to harmful exposures associated with human morbidity [1-5]. The empirical characterization of environmental conditions, however, is challenging because the non-residential ambient environment comprises an almost uncountable array of complex mixtures, which are difficult to quantify simultaneously. Moreover, the effect of the surrounding environment on human morbidity is more broadly understood to include exposures such as socioeconomic deprivation, access to healthy food, highway safety, etc. The complex nature of the overall environment likely contributes to the practice of using isolated exposures to represent ambient conditions.

Environment often encompasses traditional exposure like pollutants, chemicals, and water quality, as well as other non-genetic exposures such as the built environment, nutrition, and socioeconomic climate. In environmental epidemiology, ambient conditions are usually explored singly: one exposure or category of exposure at a time (e.g., ozone, pesticides, water disinfection by-products) [6]. Sometimes mixtures are used within one domain (e.g., air data) [1,7], and other times total environments may be characterized (e.g., exposure to healthy food environment) [8,9]. Still other work includes entire environmental domains to estimate non-residential ambient conditions (e.g., socioeconomic deprivation) [10,11]. And rarely, if ever, are multiple environmental domains combined, even though we know humans are exposed to these multiple environmental domains simultaneously.

Multiple challenges exist in combining across environmental domains or environmental types to construct one environmental measure. Much of the data we use to characterize environmental conditions are collected for administrative, regulatory and non-research purposes [12]. Measures collected at different scales would need to be meaningfully combined. They may also be measured at different units of spatial and temporal aggregation. A more complete estimation of the non-residential ambient environment may also be limited by statistical approaches and disciplinary practices. Statistical imprecision of estimates may be a concern if many variables are necessary to appropriately estimate a given domain or overall environment and a limited number of outcomes are being distributed across multiple exposure and covariate categories. From a disciplinary perspective, most research teams rarely include more than one type of exposure specialist. But many of these challenges can be readily overcome with appropriate statistical methods and interdisciplinary research teams.

Here we describe a method of constructing an environmental quality index (EQI) representing multiple domains of the non-residential ambient environment, including the air, water, land, built and sociodemographic domains. This manuscript outlines a reproducible approach to the development of the EQI that capitalizes almost exclusively on publically-available data sources.

## Methods

### Domain identification

A fuller description of the methods used for EQI construction is available in Additional file 1. We initially identified three environmental domains, air, water and land, based on selected chapters from the United States (U.S.) Environmental Protection Agency (EPA) 2008 Report on the Environment (ROE) [13]. Following consultation with the ROE, the team undertook a more extensive review to complement the domains and data sources already identified, which included the following activities: 1) identifying precise literature search terms, limits and reporting format; 2) conducting a literature review on “Environment and Infant Mortality”; 3) recording findings; 4) finalizing search terms for within-domain literature review; 5) conducting a within-domain literature review; and 6) recording findings. We chose infant mortality to be the health outcome for the literature search for several reasons: 1) infant mortality is a well-researched and understood health outcome; 2) infant mortality is a general outcome, with known positive associations with other lifetime health measures such as disability-adjusted life expectancy [14]; as such, the environmental exposure–health outcome relationship would not be restricted to one organ (e.g., heart disease) or system (e.g., asthma); 3) the research team was largely composed of reproductive/perinatal researchers for whom infant mortality was an important health outcome. The literature review was conducted in PubMed for the years 1980–2008. We added the built and sociodemographic domains based on the findings of the literature review. From this broad search, and our a priori identification, five specific domains were considered: air, water, land, built, and sociodemographic environments.

### Geographic level of analysis

The unit of analysis for EQI development was U.S. county. While county is a broad unit of analysis that may not allow for small-geography specificity, most national data sources are available at the county level. We wanted to construct a replicable process and product for use across the United States and we deemed the county level as the most widely generalizable. It also enables linkage to health data aggregated to the county level, such as national birth statistics from the National Center for Health Statistics (NCHS).

### Data source time period

At the initiation of the EQI development, we restricted the temporal framework to 2000 to 2005. We wanted to primarily utilize publicly available data, and this six-year window was chosen based on availability of both environmental (including decennial census) and outcome data (e.g., national birth records).

### Data sources

The data sources are described in detail elsewhere [15]. Briefly, data sources were considered for EQI inclusion based on temporal, spatial, and quality-related criteria. Temporal appropriateness required data to represent the 2000 to 2005 time period. Data sources were considered spatially appropriate if data were available at, or could be aggregated or interpolated to represent, the county level for all 50 states. Data quality, especially related to data source documentation, was determined by data source managers (in data reports and internal documentation), project investigators, and with the larger field of environmental research, through use and critique of the various data sources.

The air domain included two data sources: the Air Quality System (AQS) [16], which is a repository of national ambient air concentrations from monitors across the country for criteria air pollutants; and the National-Scale Air Toxics Assessment (NATA) [17], which uses emissions inventory data and air dispersion models to estimate non-residential ambient concentrations of hazardous air pollutants (HAPs).

The water domain comprised five data sources: Watershed Assessment, Tracking < Environmental Results (WATERS) Program Database [18], Estimates of Water Use in the U.S. [19], National Atmospheric Deposition Program (NDAP) [20], Drought Monitor Network [21], and National Contaminant Occurrence Database (NCOD) [22]. The WATERS Program Database is a collection of data from various EPA-conducted water assessment programs including impairment, water quality standards, pollutant discharge permits, and beach violations and closures. The Estimates of Water Use in the U.S. is calculated by the United States Geological Survey (USGS) and includes county-level estimates of water withdrawals for domestic, agricultural, and industrial uses. The NDAP dataset provides measures of chemicals in precipitation using a network of monitors located throughout the U.S. The Drought Monitor Data provides raster data on the drought status for the entire U.S. on a weekly basis. The NCOD dataset provides data from public water supplies on 69 different contaminants.

The land domain was constructed using data from five sources. The 2002 National Pesticide Use Database [23] estimates state-level pesticide usage based on pesticide ingredients and crop type. The 2002 Census of Agriculture [24] is a summary of agricultural activity, including information about crops, livestock, and chemicals used. The National Priority Site data [25] includes location of and information on sites that have been placed on the National Priority List (NPL), including indicators for major facilities (e.g., Superfund sites), large quantity generators, toxics release inventory, Resources Conservation and Recovery Act treatment, storage and disposal facilities, corrective action facilities, assessment, cleanup, and redevelopment exchange (brownfield sites), and section seven tracking system pesticide producing site locations. The National Geochemical Survey [26] contains geochemical data (e.g., arsenic, selenium, mercury, lead, zinc, magnesium, manganese, iron, etc.). The fifth source is the EPA Radon Zone Map [27], which identifies areas of the U.S. with the potential for elevated indoor radon levels.

The sociodemographic domain included two data sources: the U.S. Census [28] and Federal Bureau of Investigation (FBI) Uniform Crime Report (UCR) [29]. The U.S. Census collects population and housing data every 10 years, economic and government data every five years and the American Community Survey annually. FBI UCR rate data are available annually and by crime type (violent or property).

The built environment domain employed five data sources. Dun and Bradstreet collects commercial information on businesses and contains more than 195 million records [30]. These data are the only data used in the EQI which are not free, though they are publically available for purchase. Topographically Integrated Geocoding Encoding Reference (TIGER) [31] data provides maps and road layers for the U.S. at multiple units of census geography. The Fatality Analysis Reporting System (FARS) [32] data is a national census providing the National Highway Traffic Safety administration yearly reports of fatal injuries suffered in motor vehicle crashes. Housing and Urban Development (HUD) [33] data provide a count of low-rent and section-eight housing in each housing authority area, which corresponds to cities. The built environment domain also included the percent using public transportation variable from the census, which was not included in the sociodemographic domain; census data have been previously described.

### EQI construction

#### Variable construction

Each of the data sources could plausibly give rise to hundreds of potentially relevant variables; therefore only specific variables were selected – or in some cases, constructed – from each of the data sources. A detailed listing of all the constructed variables is available in Additional file 2.

#### Statistical processes common to all variables in all domains

Variable collinearity was assessed within subgrouping and when the correlation coefficients exceeded 0.7, one variable was chosen for inclusion. Similar variables with low numbers of missing values were retained over those with high numbers of missing values. If missingness was approximately equal, the decision about which variable to retain was based on exposure routes from hazard summaries [34], with routes from the appropriate domain being primary.

Variable missingness was also assessed to determine if missing data were missing or instead represented true zeros. For instance, when crime data was missing for a county we considered that missing because crime occurs most everywhere but when beach closure data were missing for a county, we considered those to be true zeros because not all counties have beaches. When more than 50 percent of all counties were missing or zero for a given variable, that variable was excluded from further consideration for the EQI.

Because of the data reduction approach used for index construction (principal components analysis (PCA), discussed in detail below), and the statistical assumptions implied by this method, variables were assessed for normality. This was done by visually comparing histograms of each variable’s distribution to a normal distribution for that variable. When violations of normality were observed, transformations were considered to enable the variable to best approximate the normal distribution. For each variable, natural-log (hereafter, log), logit, and squared-root transformations were considered and distributions were visually inspected again. In each case, log transformation resulted in the most normally-appearing distribution. For variables with true zeros, log-transformation was achieved by adding half of the non-zero minimum value to all observations and then taking the natural log of that value.

Finally, variables were assessed to determine valences for environmental quality. Valences, or the positive or negative direction of the indices, were determined based on potential for human health and ecological effects. Domains containing variables with known or suspected potential for adverse health outcomes (e.g., increased morbidity or mortality) or ecologic effects (e.g., disruption of biotic integrity) were considered to have a negative valence with higher values representing poorer environmental quality. In some cases, the valence of a given variable was unknown, in which case the valence would be empirically assigned through the data reduction/PCA process by virtue of its association with other variables in that domain.

#### Air domain variable construction

Daily concentrations of six criteria air pollutants were downloaded from the AQS [16] and temporally averaged to get annual mean concentrations for each monitor location from 2000 to 2005. The annual means were then temporally and spatially kriged to estimate annual concentrations at each county center point. An exponential covariance structure for the spatial covariance was implemented to represent both temporal and spatial variability. These values were then averaged for the full study period.

The 2002 NATA [17] database was used as an initial source of county-level HAP concentrations for evaluation of variables to include. Emissions estimates were retrieved from the NATAs for 1999 and 2005, and estimates for each variable from the three NATAs were averaged to get a composite emissions estimate across the study period. Air domain variables were then checked for normality of distribution and where indicated, were log-transformed. For both criteria and hazardous air pollutants, higher concentrations are negative for air quality. Therefore, the valence of the air domain is negative.

#### Water domain variable construction

Water impairment is determined for multiple types of water usage: agricultural, drinking, recreational, wildlife and industrial. Using the WATERS [18] database and joining the data in GIS software with measures of stream length in the Reach Address Database [35], a cumulative measure of percent of water impaired for any use was used to represent overall water quality in the county.

Water contamination is caused by several sources and we used the number of National Pollutant Discharge Elimination System (NPDES) [36] permits in a county as a proxy for general water contamination. Three composite variables were included in the EQI: a composite for number of sewage permits, a composite for industrial permits, and one for stormwater permits, all per 1000 km of stream length per county.

Recreational water quality was assessed also using the WATERS database [18], from which we created three variables for number of days of beach closure - for any event, for contamination events, and for rain events in a county.

The quality of the water used for domestic needs data was extracted from the Estimates of Water Use in the U.S. [19] database as a proxy for domestic water quality from which two variables were included in the EQI: the percent of population on self-supplied water supplies and the percent of those on public water supplies which are on surface waters.

The atmospheric deposition of chemicals can affect water quality. The NDAP [20] dataset provides measures for the concentration of nine chemicals in precipitation, calcuim, magnesium, potassium, sodium, ammonium, nitrate, chloride, sulfate, and mercury. Annual summary data from each monitoring site for each year 2000–2005 were spatially kriged, using an exponential covariance structure, to achieve national coverage and county level estimates. The annual estimates for each pollutant were then averaged over the six-year study period. The data for all pollutants, except sulfate, were skewed and therefore were log-transformed to achieve normal distributions.

We expect that drought affects the concentration of pathogens and chemicals in waters and therefore can affect water quality. The Drought Monitor [21] dataset provides raster data on six possible drought status conditions for the entire U.S. on a weekly basis. The data were spatially aggregated to the county level to estimate the percentage of the county in each drought status condition. From this data we used the percentage of the county in extreme drought (D3-D4) in the EQI.

Chemical contamination of water supplies was extracted from the NCOD [22] dataset which provides data on 69 contaminants provided by public water supplies throughout the country for the period from 1998–2005. Data for all samples in a county for each contaminant were averaged over the entire time period of the data and log-transformed to achieve normal distributions. Missing values were set to zero, with the assumption that lack of measurement for an area indicated low concern for contamination with that particular contaminant.

The majority of variables in the water domain are estimates of pollutants for which higher values are considered negative for water quality. The final valence of the water domain is negative, indicating a higher water domain score is associated with poorer environmental quality.

#### Land domain variable construction

Information on the agricultural environment, were obtained from the 2002 Census of Agriculture [24]. In total, eight variables representing agriculture were constructed and county-level percentages (acres applied per county total acreage) were calculated and log-transformed.

Variables specific to pesticide application were also constructed. Herbicide, insecticide, and fungicide use for each county were estimated using crop data from the 2002 Census of Agriculture and state pesticide use data from the 2002 National Pesticide Use Dataset [23]. All pesticide variables were log-transformed.

The natural geochemistry and soil contamination of an area was estimated using the National Geochemical Survey (NGS) data [26]. These data, collected for stream sediments, soils, and other media, were combined at the county level to estimate the mean values of 13 geochemical contaminants available and were log-transformed.

Large industrial facilities represent sources for pollutants released into the environment. The National Priority List [25] data from the EPA provided information on facilities for the U.S. Because many counties had at least one, but no counties had all six of the facility types present, a composite facilities data variable was constructed by summing the count of any one of the six facilities types (brownfield sites [37], superfund sites [38], toxic release inventory sites [39], pesticide producing location sites [40], large quantity generator sites [41], and treatment, storage and disposal sites [42]) across the counties. The facilities rate variable was assessed for normality and log-transformed.

Finally, the potential for elevated indoor radon levels was represented using county score from the EPA Radon Zone map [27].

As all constructs in the land domain were determined to have a negative valence, the valence of the land domain as a whole is also negative, indicating a higher land domain score represents poor environmental quality.

#### Sociodemographic domain

The sociodemographic environment is an important environment for human health. Eleven variables from the United States Census [28] were included in the sociodemographic domain of the EQI. The sociodemographic domain contains a mix of positive and negative features; therefore when the sociodemographic domain was constructed, positive variables were reverse-coded to ensure that a higher amount of the sociodemographic domain represented adverse environmental conditions.

The area-level crime environment was represented using the Federal Bureau of Investigation (FBI) Uniform Crime Reports (UCR) [29]. These data required some manipulation for inclusion in the EQI. Because crime reporting is voluntary and crime data are reported for less than half the U.S. counties, yet it seemed unlikely that no crimes occurred in the areas with no reported crime, crime data were spatially and temporally kriged to estimate values for counties with no reported crime. Kriging employed a double exponential covariance structure for the spatial covariance; one structure represented short-range variability and the other long-range variability. The covariance model was fit to experimental covariance values using a least squares method and demonstrated sufficient fit. Varying geographical unit sizes were not explicitly accounted for through the kriging estimates, but crime estimates were made for 57 percent of U.S. counties, mostly in rural areas. The crime variable was log-transformed for inclusion in the EQI.

Both constructs in the sociodemographic domain have a negative valence. Therefore, the final valence of the sociodemographic domain is negative, indicating a higher sociodemographic domain score is associated with poor environmental quality.

#### Built environment domain

Housing environments vary and features of the housing environment have the potential to influence human health and well-being. The housing environment was represented using two variables available from the HUD data source, low-rent and section-eight [43], which were summed to result in the count of any low-rent or section-eight housing in each county; the subsidized housing rate was constructed from this count. The subsidized housing rate was log-transformed.

Highway safety was represented by a traffic fatality variable. Rates for the count of fatal crashes per county were constructed. This rate was log distributed (due to many counties having zero fatal crashes) and was therefore log-transformed. The percent of county residents who use public transportation was the only U.S. Census [28] variable used in the built environment domain of the EQI. For many counties, the percent of the population who reports using public transportation is near 0, and it was therefore log-transformed.

We were interested in characterizing the relative proportions of each county that were served by highways, secondary roads and primary roads. The proportions of all roadways that were highways or primary roads were included.

Business and service environments are important predictors of human health and activity. We sought to estimate features of the economic and service environment using data from Duns and Bradstreet [30]. Nine business environment rate variables were constructed by dividing the county-level count of a business type by the county-level population count. All variables except the negative food environment were log-transformed for normality. The business and service environments contain a mix of positive and negative features; therefore when the built domain was constructed, positive variables were reverse-coded to ensure that a higher amount of the these service variables represent adverse environmental conditions. The built domain’s valence is negative indicating a higher built domain score represents poor environmental quality.

#### EQI temporal representation

When annual data were available, variable consistency (mean and standard deviation) was compared across each year of the six-years (2000–2005). Additionally, proto-EQIs were constructed using data from one year (2002) and from the average of all six-years. For those variables that were spatially kriged, county-level values before and after kriging were also compared. Because these county-level values were temporally consistent, the EQI was constructed based on county-level averages for the six-year period for each variable in each domain.

#### RUCC stratification

Recognizing that environments differ across the rural–urban continuum [44], we concluded the EQI would be most useful if it accommodated rural–urban environmental differences. Therefore, EQI construction was stratified by rural–urban continuum codes (RUCC). The RUCC is a nine-item categorization code of proximity to/influence of major metropolitan areas [45]. As has been done elsewhere, the nine-item categories were condensed into four categories for which RUCC1 represents metropolitan urbanized = codes 1 + 2 + 3; RUCC2 non-metro urbanized = 4 + 5; RUCC3 less urbanized = 6 + 7; and RUCC4 thinly populated =8 + 9 [46-49]. Both stratified county-specific and all-county indices were created. Loadings on the stratified and non-stratified sets of indices were assessed to determine loading heterogeneity across counties. Because these loadings differed meaningfully by RUCC level, we constructed a RUCC-stratified EQI for each county.

#### Data reduction

Similar to the approach employed in other research [10,50,51], principal components analysis (PCA) was chosen for data reduction in this study because the investigators sought an empirical summary of total area-level variance explained by the environmental variables, rather than a confirmation of any underlying factor structure comprised of the previously identified domains.

Because it was unclear which of the variables included in the domain-specific PCAs were irrelevant to human health, we retained all the variables for inclusion in the RUCC-stratified and overall indices.

#### Component extraction and index construction

The constructed variables from each dataset were merged to produce a domain-specific county-level dataset. The domain-specific variables were then combined using PCA. PCA produces variable loadings, which are roughly equivalent to the “weight” or contribution that each variable makes toward explaining the total variance. The loading associated with each variable is then multiplied by its mean value for the given geography (county, for the EQI) and these weighted mean values are summed. Although it is possible to form as many independent linear combinations as there are variables, we retained only the first principal component: the unique linear combination that accounted for the largest possible proportion of the total variability in the component measures. This process was undertaken separately for each of the four RUCC strata.

**Figure 1:**
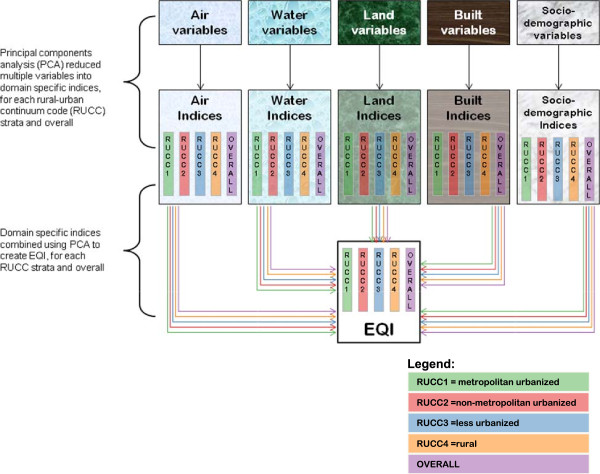
Domain-specific and overall EQI construction - conceptually.

The first principal component, which we labeled the domain-specific index (e.g., air domain index), was standardized to have a mean of 0 and standard deviation (SD) of 1 by dividing the index by the square of the eigenvalue [52]. Each domain-specific index was then included in a second PCA procedure (Figure 1), from which we extracted the first principal component to create the EQI.

Pearson’s product moment correlations were used to assess relationships among the indices and between the indices and other county-level variables with a cut off of 0.7.

## Results

### Description of variables comprising EQI domains

The full listing and description of variables contained in the EQI can be found in Additional file 2. Here we present exemplar variables from each domain to describe the variables that represented common patterns of variable loadings (e.g., monotonically increasing or decreasing loadings from most urban to most rural, u-shaped loading pattern from most urban to most rural, etc.). Means, standard deviations, and ranges are included.

Variables included in the air domain generally show moderate to high variability between rural–urban strata, with higher averages in the most urban stratum decreasing to the most rural stratum (Table 1). For example, CO has mean values of 705, 598, 472, 343 ppm for each stratum from most urban to most rural. This pattern holds true for most of the hazardous air pollutants as well, though some pollutants show higher means in the non-metro urbanized or less-urbanized strata (e.g., chlorine, dimethyl sulfate). PM_10_, PM_2.5_, and carbon tetrachloride are relatively stable across rural–urban strata.

**Table 1:** Overall and RUCC-stratified domain variable means, standard deviations, ranges for select variables in the air domain

		**Metropolitan - urbanized**	**Non-metro urbanized**	**Less-urbanized**	**Thinly populated**	**Overall**
		**(RUCC1 = 1089)**	**(RUCC2 = 323)**	**(RUCC 3 = 1059)**	**(RUCC4 = 670)**	**(n = 3141)**
**Variable (construct)**	**Units**	**Mean (sd) [range]**	**Mean (sd) [range]**	**Mean (sd) [range]**	**Mean (sd) [range]**	**Mean (sd) [range]**
Nitrogen Dioxide	Ppb	7.95E + 02 (7.05E + 02)	4.97E + 02 (4.13E + 02)	4.21E + 02 (3.95E + 02)	3.53E + 02 (3.36E + 02)	5.44E + 02 (5.49E + 02)
(Criteria air pollutants)		[1.01E + 00, 8.65E + 03]	[1.29E + 00, 2.59E + 03]	[1.00E + 00, 8.66E + 03]	[1.00E + 00, 3.42E + 03]	[1.00E + 00, 8.66E + 03]
PM_10_	μg/m^3^	14.199 (5.193)	11.258 (4.533)	9.852 (3.924)	8.446 (3.596)	11.204 (4.974)
(Criteria air pollutants)		[1.777, 39.554]	[2.370, 27.095]	[1.000, 34.625]	[1.011, 21.404]	[1.000, 39.554]
PM_2.5_	μg/m^3^	10.621 (2.205)	9.586 (2.351)	9.379 (2.466)	8.265 (2.745)	9.593 (2.582)
(Criteria air pollutants)		[3.443, 16.912]	[2.162, 14.397]	[1.029, 14.451]	[1.138, 13.437]	[1.029, 16.912]
Carbon disulfide	tons emitted	9.05E-03 (1.05E-01)	5.94E-03 (6.12E-02)	2.07E-03 (2.48E-02)	7.92E-04 (1.13E-02)	4.61E-03 (6.66E-02)
(Hazardous air pollutants)		[5.50E-07, 2.30E + 00]	[1.91E-06, 1.02E + 00]	[1.34E-07, 5.66E-01]	[5.88E-09, 2.62E-01]	[5.88E-09, 2.30E + 00]
Carbon tetrachloride	tons emitted	0.497 (0.006)	0.497 (0.003)	0.497 (0.005)	0.496 (0.008)	0.497 (0.006)
(Hazardous air pollutants)		[0.429, 0.558]	[0.468, 0.511]	[0.429, 0.568]	[0.395, 0.509]	[0.395, 0.568]
Cyanide compounds	tons emitted	3.88E-02 (5.55E-02)	0.028 (0.037)	1.29E-02 (3.04E-02)	4.05E-03 (5.00E-03)	2.16E-02 (4.16E-02)
(Hazardous air pollutants)		[4.91E-04, 1.35E + 00]	[0.003, 0.635]	[1.15E-04, 9.50E-01]	[3.08E-05, 6.45E-02]	[3.08E-05, 1.35E + 00]
Diesel engine emissions	tons emitted	0.607 (0.516)	0.352 (0.188)	0.235 (0.129)	1.53E-01 (9.87E-02)	3.59E-01 (3.73E-01)
(Hazardous air pollutants)		[0.034, 8.815]	[0.035, 1.791]	[0.001, 0.991]	[1.59E-04, 5.42E-01]	[1.59E-04, 8.82E + 00]
Lead compounds	tons emitted	2.23E-03 (3.84E-03)	1.43E-03 (1.65E-03)	9.34E-04 (2.21E-03)	6.01E-04 (1.48E-03)	1.36E-03 (2.82E-03)
(Hazardous air pollutants)		[3.41E-04, 8.17E-02]	[3.51E-04, 1.45E-02]	[2.98E-04, 4.74E-02]	[2.74E-04, 2.35E-02]	[2.74E-04, 8.17E-02]
PAH/POM	tons emitted	1.62E-02 (3.00E-02)	0.014 (0.021)	7.66E-03 (1.58E-02)	3.20E-03 (7.15E-03)	1.03E-02 (2.19E-02)
(Hazardous air pollutants)		[1.79E-04, 4.45E-01]	[0.001, 0.139]	[2.64E-05, 3.36E-01]	[4.44E-05, 1.25E-01]	[2.64E-05, 4.45E-01]
Polychlorinated biphenyls	tons emitted	1.71E-04 (4.16E-05)	1.84E-04 (3.62E-05)	1.69E-04 (2.06E-04)	1.43E-04 (6.60E-05)	1.66E-04 (1.27E-04)
(Hazardous air pollutants)		[1.27E-04, 5.94E-04]	[1.27E-04, 3.47E-04]	[1.27E-04, 6.27E-03]	[1.27E-04, 1.59E-03]	[1.27E-04, 6.27E-03]
Vinyl chloride	tons emitted	1.10E-02 (1.09E-02)	6.93E-03 (3.54E-03)	1.33E-03 (3.75E-03)	1.60E-04 (1.09E-03)	4.99E-03 (8.36E-03)
(Hazardous air pollutants)		[5.00E-06, 1.05E-01]	[1.27E-06, 1.97E-02]	[4.37E-09, 9.41E-02]	[6.99E-10, 2.56E-02]	[6.99E-10, 1.05E-01]

Complete list of variables is available in Additional file 2.

The variables included in the water domain also demonstrate moderate variability across the rural–urban strata. The metropolitan-urbanized and non-metro urbanized strata both have higher overall impaired stream length (14.00% and 14.20%, respectively) compared to the less-urbanized and thinly populated strata (8.79% and 6.54% respectively) (Table 2). The urban strata also demonstrated a higher number of discharge permits per stream length than the rural strata. The thinly-populated stratum had the highest percentage of population on self-supplied sources (35.61%) and the lowest percentage of population on surface water sources (21.94%). While most chemical contaminants demonstrated similar concentrations across the rural–urban strata, there were a few differences. Fluoride and Di(2-ethylhexyl)adipate (DEHA) were present in higher concentrations on the metropolitan-urbanized stratum. There was little variability across rural–urban strata for atmospheric deposition of chemicals and percent of land in extreme drought.

**Table 2:** Overall and RUCC-stratified domain variable means, standard deviations, ranges for select variables in the water domain

		**Metropolitan - urbanized**	**Non-metro urbanized**	**Less-urbanized**	**Thinly populated**	**Overall**
		**(RUCC1 = 1089)**	**(RUCC2 = 323)**	**(RUCC 3 = 1059)**	**(RUCC4 = 670)**	**(n = 3141)**
**Variable (construct)**	**Units**	**Mean (sd) [range]**	**Mean (sd) [range]**	**Mean (sd) [range]**	**Mean (sd) [range]**	**Mean (sd) [range]**
Impaired stream length in county	percent	14.004 (16.528)	14.203 (20.403)	8.791 (12.682)	6.537 (9.470)	10.674 (14.853)
(Overall water quality)		[0, 92.570]	[0, 94.450]	[0, 95.740]	[0, 98.500]	[0, 98.500]
Industrial Permits	permits/1000 km	51.139 (96.466)	27.241 (31.332)	18.423 (38.732)	10.080 (22.009)	28.893 (64.947)
(General water contamination)		[0, 1195.680]	[0, 280.860]	[0, 674.150]	[0, 337.300]	[0, 1195.680]
Number of days of beach closure	days	3.229 (19.625)	1.421 (9.859)	0.143 (1.945)	0.022 (0.508)	1.318 (12.118)
(Recreational water quality)		[0, 365.000]	[0, 116.000]	[0, 55.000]	[0, 13.000]	[0, 365.000]
Percent of Public Supply Population on Surface Water	percent	46.863 (41.736)	41.922 (41.306)	33.775 (40.841)	21.942 (36.526)	36.627 (41.386)
(Domestic use water quality)		[0, 100.000]	[0, 100.000]	[0, 100.000]	[0, 100.000]	[0, 100.000]
Calcium (Ca) precipitation weighted mean	mg/L	0.192 (0.120)	0.217 (0.130)	0.255 (0.144)	0.279 (0.144)	0.231 (0.139)
(Atmospheric deposition)		[0.040, 0.594]	[0.043, 0.634]	[0.042, 1.183]	[0.047, 0.806]	[0.040, 1.183]
Total Mercury (Hg) deposition	ng/M2	4.784 (1.249)	4.635 (1.364)	4.780 (1.397)	4.520 (1.405)	4.711 (1.350)
(Atmospheric deposition)		[1.101, 9.219]	[1.100, 7.950]	[1.109, 8.473]	[1.103, 8.458]	[1.100, 9.219]
Percent of county drought – extreme (D3-D4)	percent	3.160 (5.273)	3.522 (6.215)	3.908 (6.931)	5.030 (8.577)	3.848 (6.777)
(Drought)		[0, 46.900]	[0, 42.430]	[0, 40.400]	[0, 48.800]	[0, 48.800]
Alpha Particles	PCl/L	1.034 (2.333)	1.113 (1.851)	1.364 (3.517)	0.781 (2.053)	1.099 (2.711)
(Chemical contamination)		[0, 35.800]	[0, 11.390]	[0, 51.450]	[0, 18.100]	[0, 51.450]
Selenium	mg/L	3.13E-03 (4.73E-03)	2.93E-03 (3.65E-03)	3.00E-03 (5.12E-03)	2.25E-03 (4.45E-03)	4.72E-03 (2.88E-03)
(Chemical contamination)		[0, 5.00E-02]	[0, 3.00E-02]	[0, 9.40E-02]	[0, 4.70E-02]	[0, 9.40E-02]
Silvex	ug/L	0.384 (1.020)	0.579 (1.836)	0.384 (1.087)	0.184 (0.633)	0.362 (1.096)
(Chemical contamination)		[0, 5.000]	[0, 25.250]	[0, 12.500]	[0, 5.000]	[0, 25.250]
Chlordane	ug/L	0.088 (0.100)	0.099 (0.096)	0.090 (0.097)	0.068 (0.094)	0.086 (0.098)
(Chemical contamination)		[0, 0.950]	[0, 0.273]	[0, 0.267]	[0, 0.200]	[0, 0.950]
Tetrachloroethylene	ug/L	0.460 (0.584)	0.397 (0.376)	0.407 (0.771)	0.325 (0.300)	0.407 (0.595)
(Chemical contamination)		[0, 8.000]	[0, 5.110]	[0, 23.750]	[0, 4.330]	[0, 23.750]
1,2-Dichloropropane	ug/L	0.360 (0.232)	0.364 (0.243)	0.368 (0.217)	0.313 (0.240)	0.353 (0.231)
(Chemical contamination)		[0, 1.270]	[0, 1.900]	[0, 0.560]	[0, 0.620]	[0, 1.900]

Complete list of variables is available in Additional file 2.

In the land domain, the metropolitan-urbanized counties have higher averages of soil contaminants, more facilities, and lower agricultural-related variables (% harvested,% irrigated) than non-metro urbanized, less-urban, and thinly-populated counties (Table 3). Pesticides and animal units show no clear pattern in variation across the strata. For example, average pounds of fungicides applied are 1820, 4030, 2740, and 2140 for most urban to most rural strata, respectively. There is little variation in the distribution of radon zones or agricultural chemicals applied across rural–urban strata.

**Table 3:** Overall and RUCC-stratified domain variable means, standard deviations, ranges for select variables in the land domain

		**Metropolitan - urbanized**	**Non-metro urbanized**	**Less-urbanized**	**Thinly populated**	**Overall**
		**(RUCC1 = 1089)**	**(RUCC2 = 323)**	**(RUCC 3 = 1059)**	**(RUCC4 = 670)**	**(n = 3141)**
**Variable (construct)**	**Units**	**Mean (sd) [range]**	**Mean (sd) [range]**	**Mean (sd) [range]**	**Mean (sd) [range]**	**Mean (sd) [range]**
Harvested acreage	acres harvested per county acres	0.183 (0.208)	0.240 (0.244)	0.240 (0.253)	0.201 (0.218)	0.212 (0.231)
(Agriculture)		[0, 0.920]	[0, 0.895]	[0, 1.221]	[0, 0.946]	[0, 1.221]
Animal units	animal units per county acres	0.151 (0.727)	0.079 (0.116)	0.174 (1.639)	0.103 (0.143)	0.141 (1.047)
(Agriculture)		[0, 20.984]	[0, 1.235]	[0, 46.941]	[0, 1.481]	[0, 46.941]
Herbicides	pounds applied	6.85E + 04 (1.28E + 05)	1.08E + 05 (1.66E + 05)	9.56E + 04 (1.52E + 05)	6.81E + 04 (1.08E + 05)	8.16E + 04 (1.38E + 05)
(Pesticides)		[0, 1.18E + 06]	[0, 1.08E + 06]	[0, 1.15E + 06]	[0, 7.16E + 05]	[0, 1.18E + 06]
Insecticides	pounds applied	3.69E + 03 (8.89E + 03)	6.14E + 03 (1.36E + 04)	5.11E + 03 (1.05E + 04)	2.75E + 03 (5.33E + 03)	4.22E + 03 (9.52E + 03)
(Pesticides)		[0, 1.72E + 05]	[0, 1.89E + 05]	[0, 1.53E + 05]	[0, 4.87E + 04]	[0, 1.89E + 05]
Arsenic	ppm	6.530 (5.445)	6.850 (8.171)	6.435 (5.139)	6.342 (4.304)	6.491 (5.476)
(Contaminants)		[0, 91.333]	[0, 131.369]	[0, 98.893]	[0, 43.595]	[0, 131.369]
Lead	ppm	29.901 (45.950)	22.049 (17.221)	21.219 (24.976)	22.812 (51.740)	24.654 (39.465)
(Contaminants)		[0, 1007.300]	[0, 196.867]	[0, 691.838]	[0, 1123.110]	[0, 1123.110]
Titanium	% weight	0.404 (0.228)	0.369 (0.185)	0.327 (0.165)	0.318 (0.185)	0.356 (0.198)
(Contaminants)		[0, 2.118]	[0, 1.405]	[0, 2.109]	[0, 1.941]	[0, 2.118]
Iron	% weight	2.531 (1.480)	2.372 (1.282)	2.152 (1.186)	2.154 (1.054)	2.307 (1.292)
(Contaminants)		[0, 13.731]	[0, 8.440]	[0, 9.461]	[0, 7.165]	[0, 13.731]
Phosphorus	% weight	0.067 (0.139)	0.056 (0.095)	0.051 (0.059)	0.053 (0.040)	0.057 (0.096)
(Contaminants)		[0, 2.203]	[0, 1.296]	[0, 1.025]	[0, 0.509]	[0, 2.203]
Facilities	facilities per county pop	3.94E-04 (2.77E-04)	4.95E-04 (3.08E-04)	5.59E-04 (4.60E-04)	7.77E-04 (2.33E-03)	5.42E-04 (1.13E-03)
(facilities)		[0, 2.30E-03]	[4.23E-05, 2.19E-03]	[0, 7.55E-03]	[0, 5.42E-02]	[0, 5.42E-02]
Radon zone	radon zone	2.010 (0.815)	2.000 (0.856)	2.022 (0.834)	1.849 (0.809)	1.979 (0.827)
(Radon)		[0, 3.000]	[0, 3.000]	[0, 3.000]	[0, 3.000]	[0, 3.000]

Complete list of variables is available in Additional file 2.

Socioeconomic variables included in the sociodemographic domain indicate that rural counties are generally more deprived than more urban counties (Table 4), having the lowest household income ($30,350) and highest percent of persons in poverty (16.1%). From the crime perspective, however, rural areas are at an advantage compared to more urban areas; the mean violent crime rate for rural counties was 352.5 compared with 390.9 for the most urban and 398.1 for the non-metropolitan urbanized counties.

**Table 4:** Overall and RUCC-stratified domain variable means, standard deviations, ranges for select variables in the sociodemographic domain

		**Metropolitan - urbanized**	**Non-metro urbanized**	**Less-urbanized**	**Thinly populated**	**Overall**
		**(RUCC1 = 1089)**	**(RUCC2 = 323)**	**(RUCC 3 = 1059)**	**(RUCC4 = 670)**	**(n = 3141)**
**Variable (construct)**	**Units**	**Mean (sd) [range]**	**Mean (sd) [range]**	**Mean (sd) [range]**	**Mean (sd) [range]**	**Mean (sd) [range]**
Renter occupied (Socioeconomic status (SES))	percent	27.734 (9.557)	29.307 (6.499)	25.338 (5.588)	22.947 (6.814)	26.067 (7.791)
		[10.561, 80.458]	[13.562, 52.731]	[13.545, 72.205]	[10.464, 100]	[10.464, 100]
Vacant units (SES)	percent	9.146 (5.810)	12.026 (7.190)	15.324 (8.392)	21.980 (11.880)	14.263 (9.668)
		[1.539, 53.707]	[3.457, 58.416]	[4.336, 62.316]	[4.183, 77.014]	[1.539, 77.014]
Median household value (SES)	dollar value	1.10E + 05 (5.51E + 04)	8.86E + 04 (3.48E + 04)	7.25E + 04 (3.90E + 04)	6.09E + 04 (3.06E + 04)	8.46E + 04 (4.77E + 04)
		[3.46E + 04, 1.00E + 06]	[3.78E + 04, 3.69E + 05]	[2.26E + 04, 7.50E + 05]	[0, 3.58E + 05]	[0, 1.00E + 06]
Median household income (SES)	dollar value	4.17E + 04 (9.84E + 03)	3.53E + 04 (6.39E + 03)	3.21E + 04 (6.03E + 03)	3.03E + 04 (5.59E + 03)	3.54E + 04 (8.92E + 03)
		[1.98E + 04, 8.29E + 04]	[1.65E + 04, 6.27E + 04]	[1.63E + 04, 7.90E + 04]	[9.33E + 03, 5.37E + 04]	[9.33E + 03, 8.29E + 04]
Persons < poverty level (SES)	percent	11.567 (5.307)	14.187 (6.275)	15.601 (6.565)	16.147 (7.107)	14.173 (6.554)
		[2.100, 35.900]	[4.500, 50.900]	[2.900, 52.300]	[0, 56.900]	[0, 56.900]
No English (SES)	percent	9.490 (10.534)	9.257 (12.094)	8.451 (12.103)	6.791 (9.492)	8.540 (11.092)
		[1.000, 91.900]	[1.900, 92.100]	[0.700, 84.800]	[0.400, 85.400]	[0.400, 92.100]
Earning > high school education (SES)	percent	80.181 (7.546)	78.708 (7.814)	74.877 (8.813)	76.139 (9.478)	77.379 (8.755)
		[50.500, 97.000]	[34.700, 93.800]	[43.400, 96.300]	[39.500, 94.400]	[34.700, 97.000]
Unemployed (SES)	percent	5.293 (2.301)	6.433 (2.468)	6.298 (2.757)	5.631 (3.745)	5.821 (2.868)
		[1.700, 41.700]	[2.500, 20.900]	[1.400, 33.000]	[0, 41.400]	[0, 41.700]
Work outside county (SES)	percent	40.137 (20.673)	21.479 (12.061)	28.042 (13.447)	32.952 (16.543)	32.608 (17.936)
		[1.100, 90.800]	[1.300, 60.800]	[0.600, 77.200]	[0, 76.400]	[0, 90.800]
Median number rooms per house (SES)	count	5.522 (0.459)	5.372 (0.345)	5.361 (0.360)	5.368 (0.485)	5.420 (0.430)
		[3.100, 7.300]	[4.000, 6.600]	[3.300, 6.400]	[2.000, 6.500]	[2.000, 7.300]
Housing with > 10 units (SES)	percent	6.856 (7.239)	4.845 (3.542)	2.689 (2.717)	1.475 (2.146)	4.096 (5.268)
		[0, 90.100]	[0.600, 35.100]	[0, 42.100]	[0, 31.400]	[0, 90.100]
Mean number of violent crimes per capita (Crime)	rate	390.884 (322.135)	397.099 (374.610)	366.303 (233.612)	352.528 (158.067)	375.054 (272.635)
		[0, 2481.800]	[0, 1955.500]	[0, 1897.300]	[0, 783.402]	[0, 2481.800]

Complete list of variables is available in Additional file 2.

Contributing to the built environment domain (Table 5), the most rural counties have the smallest proportion of highways and significantly higher rate of traffic fatalities compared with more urban areas. Urban counties had fewer education-related businesses, positive food establishments, recreation-related resources and subsidized housing units per person compared with more rural counties.

**Table 5:** Overall and RUCC-stratified domain variable means, standard deviations, ranges for select variables in the built environment domain

		**Metropolitan - urbanized**	**Non-metro urbanized**	**Less-urbanized**	**Thinly populated**	**Overall**
		**(RUCC1 = 1089)**	**(RUCC2 = 323)**	**(RUCC 3 = 1059)**	**(RUCC4 = 670)**	**(n = 3141)**
**Variable (construct)**	**Units**	**Mean (sd) [range]**	**Mean (sd) [range]**	**Mean (sd) [range]**	**Mean (sd) [range]**	**Mean (sd) [range]**
Roads that are highways	mile proportion	0.045 (0.026)	0.045 (0.025)	0.039 (0.029)	0.029 (0.031)	0.039 (0.029)
(Road system)		[0, 0.156]	[0, 0.158]	[0, 0.210]	[0, 0.291]	[0, 0.291]
Roads that are primary streets	mile proportion	0.171 (0.059)	0.148 (0.067)	0.136 (0.063)	0.119 (0.063)	0.146 (0.065)
(Road system)		[0.009, 0.536]	[0.015, 0.438]	[0, 0.406]	[0, 0.371]	[0, 0.536]
Traffic fatality rate	fatalities per co. pop	4.72E-04 (3.92E-04)	5.14E-04 (2.63E-04)	6.94E-04 (5.48E-04)	9.45E-04 (1.33E-03)	6.52E-04 (7.57E-04)
(Road safety)		[0, 5.04E-03]	[0, 1.60E-03]	[0, 6.29E-03]	[0, 1.10E-02]	[0, 1.10E-02]
Population using public transport	percent	1.699 (4.542)	0.714 (1.033)	0.447 (0.780)	0.393 (0.603)	0.897 (2.809)
(Public transit behavior)		[0, 59.600]	[0, 8.800]	[0, 10.600]	[0, 6.900]	[0, 59.600]
Vice-related	count per county population	3.56E-04 (2.10E-04)	4.48E-04 (2.29E-04)	4.71E-04 (3.32E-04)	7.25E-04 (6.56E-04)	4.76E-04 (3.98E-04)
(Business environment)		[1.66E-05, 1.96E-03]	[3.05E-05, 1.39E-03]	[2.47E-05, 2.06E-03]	[3.71E-05, 4.66E-03]	[1.66E-05, 4.66E-03]
Entertainment-related	count per county population	4.06E-04 (2.40E-04)	4.43E-04 (2.26E-04)	3.99E-04 (2.98E-04)	5.24E-04 (6.01E-04)	4.28E-04 (3.51E-04)
(Business environment)		[3.80E-05, 2.51E-03]	[6.72E-05, 1.63E-03]	[2.82E-05, 2.97E-03]	[5.15E-05, 6.80E-03]	[2.82E-05, 6.80E-03]
Education-related	count per county population	5.80E-04 (3.19E-04)	6.09E-04 (3.98E-04)	6.11E-04 (4.37E-04)	6.06E-04 (4.50E-04)	5.99E-04 (3.97E-04)
(Business environment)		[7.30E-05, 3.25E-03]	[1.01E-04, 3.33E-03]	[4.73E-05, 3.92E-03]	[6.10E-05, 3.26E-03]	[4.73E-05, 3.92E-03]
Negative food related	count per county population	7.67E-04 (2.15E-04)	8.67E-04 (2.12E-04)	8.85E-04 (2.89E-04)	8.27E-04 (5.03E-04)	8.30E-04 (3.20E-04)
(Business environment)		[9.44E-05, 2.26E-03]	[2.01E-04, 1.82E-03]	[1.35E-04, 2.82E-03]	[6.18E-05, 5.38E-03]	[6.18E-05, 5.38E-03]
Positive food related	count per county population	1.70E-03 (5.98E-04)	1.84E-03 (4.70E-04)	1.85E-03 (6.51E-04)	1.98E-03 (1.11E-03)	1.82E-03 (7.50E-04)
(Business environment)		[3.88E-04, 1.04E-02]	[6.28E-04, 4.63E-03]	[3.82E-04, 7.87E-03]	[1.92E-04, 1.49E-02]	[1.92E-04, 1.49E-02]
Health care related	count per county population	2.69E-03 (1.39E-03)	2.96E-03 (8.53E-04)	2.56E-03 (9.80E-04)	2.15E-03 (1.03E-03)	2.56E-03 (1.16E-03)
(Business environment)		[1.94E-04, 2.47E-02]	[7.79E-04, 8.89E-03]	[1.42E-04, 1.13E-02]	[1.42E-04, 7.66E-03]	[1.42E-04, 2.47E-02]
Recreation related	count per county population	2.49E-04 (1.24E-04)	3.11E-04 (1.47E-04)	3.32E-04 (2.41E-04)	5.13E-04 (6.47E-04)	3.30E-04 (3.29E-04)
(Business environment)		[3.38E-05, 1.16E-03]	[2.91E-05, 1.13E-03]	[3.03E-05, 2.00E-03]	[5.57E-05, 1.08E-02]	[2.91E-05, 1.08E-02]
Social service related	count per county population	8.64E-05 (5.72E-05)	9.18E-05 (5.12E-05)	1.17E-04 (8.79E-05)	2.03E-04 (1.54E-04)	1.10E-04 (9.03E-05)
(Business environment)		[9.77E-06, 7.06E-04]	[6.79E-06, 2.76E-04]	[1.44E-05, 8.37E-04]	[2.97E-05, 1.06E-03]	[6.79E-06, 1.06E-03]
Total subsidized units	count per county population	1.11E-02 (2.91E-02)	1.27E-02 (1.82E-02)	0.010 (0.019)	0.009 (0.042)	1.03E-02 (2.86E-02)
(Subsidized housing environment)		[0, 6.46E-01]	[0, 1.77E-01]	[0, 0.416]	[0, 0.834]	[0, 8.34E-01]

Complete list of variables is available in Additional file 2.

### Variable loadings on EQI domains

Variable loadings are a function of the county-level prevalence of a variable and its association with the other variables contributing to the total county-level variability for a given domain. The full listing variable loadings across RUCC strata and on the overall EQI can be found in Additional file 3. Here we present exemplar variables from each domain to describe the variables that represented common patterns of variable loadings (e.g., monotonically increasing or decreasing loadings from most urban to most rural; u-shaped loading pattern from most urban to most rural, etc.).

The loadings for the variables that comprise the air domain varied by RUCC strata, though not extensively (Table 6). Direction of loadings were similar across rural–urban strata. Criteria air pollutants were less influential in the metropolitan-urban stratum compared to the other strata, while influence of hazardous air pollutants varied. The first principal component explained 47% of the total air variability and the domain was approximately normally distributed.

**Table 6:** Overall and RUCC-stratified loadings for select variables in the air domain

**Constructs indented**	**Metropolitan - urbanized**	**Non-metro urbanized**	**Less-urbanized**	**Thinly populated**	**Overall**
	**(RUCC1 = 1089)**	**(RUCC2 = 323)**	**(RUCC 3 = 1059)**	**(RUCC4 = 670)**	**(n = 3141)**
**Air Domain**					
Criteria air pollutants					
Nitrogen Dioxide	0.0613	0.1091	0.1014	0.0911	0.0848
PM_10_	0.0845	0.0677	0.0627	0.0937	0.0897
PM_2.5_	0.0701	0.1513	0.1281	0.1354	0.1036
Hazardous air pollutants					
Carbon disulfide	0.1169	0.1140	0.1172	0.1261	0.1242
Carbon tetrachloride	0.0259	0.0281	0.0186	−0.0028	0.018
Cyanide compounds	0.1655	0.1674	0.1680	0.1497	0.1477
Diesel engine emissions	0.1545	0.1441	0.1431	0.1163	0.1321
Dimethyl sulfate	0.0472	0.1201	0.1024	0.1072	0.0942
Lead compounds	0.1366	0.0778	0.069	0.0581	0.1045
PAH/POM	0.1143	0.0822	0.1172	0.1192	0.1199
Polychlorinated biphenyls	0.0284	0.1300	0.0954	0.0779	0.0729
Vinyl chloride	0.1489	0.0997	0.1008	0.097	0.1257

Complete list of variable loadings is available in Additional file 3.

The loadings for the variables that comprise the water domain varied by RUCC and also by construct, suggesting that some constructs were more influential in urban areas and others in rural areas (Table 7). Variables representing overall water quality loaded positively in the two urban RUCC and negatively in the rural RUCC strata. The loadings for variables representing general water contamination and recreational water quality varied by RUCC though they were overall quite low. Loadings for variables representing domestic water quality and drought varied by RUCC, though they were all positive. The loadings for variables representing the atmospheric deposition construct varied by RUCC and did not demonstrate any clear patterns. Variables in the chemical contamination construct demonstrated little variability by RUCC with loadings of similar values for all variables across all RUCC. The first principal component explained 46% of the total variability for the water variables, and while each of the variables contributing to the water domain were normally distributed, the water domain itself was not. This may have resulted from so many regions of the U.S. lacking water quality information; there was considerable data for some counties and almost no data for others. In light of its non-normal distribution, the water domain itself and its contribution to the overall EQI should be interpreted with caution.

**Table 7:** Overall and RUCC-stratified loadings for select variables in the water domain

**Constructs indented**	**Metropolitan - urbanized**	**Non-metro urbanized**	**Less-urbanized**	**Thinly populated**	**Overall**
	**(RUCC1 = 1089)**	**(RUCC2 = 323)**	**(RUCC 3 = 1059)**	**(RUCC4 = 670)**	**(n = 3141)**
**Water Domain**					
Overall water quality					
% of stream length impaired in county	0.0078	0.0063	−0.0067	−0.0172	0.0031
General water contamination					
Industrial permits	−0.0214	−0.0394	−0.0078	0.0084	−0.0114
Recreational water quality					
# of days of beach closure	−0.0019	−0.0072	0.0085	0.0092	0
Domestic use water quality					
% Population which is on surface water supply	0.0190	0.0175	0.0098	0.0346	0.0220
Atmospheric deposition					
Calcium (Ca) precipitation weighted mean	0.0325	0.0179	0.0231	−0.0055	0.0154
Total Mercury (Hg) deposition	−0.0413	−0.0359	−0.0293	0.0072	−0.0228
Drought					
% of county drought – extreme (D3-D4)	0.0035	0.0337	0.0234	0.0242	0.0164
Chemical contamination					
Selenium	0.1123	0.1196	0.1044	0.1071	0.1103
Silvex	0.1217	0.1209	0.1204	0.1270	0.1226
Chlordane	0.1314	0.1344	0.1343	0.1346	0.1336
1,2-Dichloropropane	0.1451	0.1452	0.1512	0.1461	0.1473
Alpha Particles	0.0620	0.0794	0.0703	0.0704	0.0691

Complete list of variable loadings is available in Additional file 3.

The loadings for variables in the land domain varied considerably (Table 8). For mercury, lead, titanium, and aluminum, loading magnitudes were much lower in the most urban stratum, while the loadings across all other strata were comparable. Some variables had the highest loading in the most-urban and most-rural strata (e.g., herbicides), while others remained stable across strata (e.g., arsenic, iron, harvested acreage). Direction of loadings was consistent across strata and the first principal component accounted for 32% of the total variability. This domain was approximately normally distributed with just a few counties having significantly lower land-domain values. These outlying counties were retained, however, to enable the EQI’s construction for all U.S. counties.

**Table 8:** Overall and RUCC-stratified loadings for select variables in the land domain

**Constructs indented**	**Metropolitan - urbanized**	**Non-metro urbanized**	**Less-urbanized**	**Thinly populated**	**Overall**
	**(RUCC1 = 1089)**	**(RUCC2 = 323)**	**(RUCC 3 = 1059)**	**(RUCC4 = 670)**	**(n = 3141)**
**Land Domain**					
Agriculture					
Harvested acreage	0.1572	0.1395	0.1398	0.1373	0.1400
Animal units	−0.0358	−0.0133	0.0019	0.0202	−0.0034
Pesticides					
Herbicides	0.1701	0.1393	0.1350	0.1762	0.1524
Insecticides	0.1407	0.1049	0.0874	0.1047	0.1072
Contaminants					
Arsenic	0.2617	0.2774	0.2722	0.2626	0.2685
Lead	0.1731	0.2300	0.2395	0.2386	0.2228
Titanium	0.1012	0.1965	0.1701	0.2020	0.1682
Iron	0.3099	0.3218	0.3139	0.2948	0.3144
Phosphorus	0.1011	0.0858	0.1428	0.1775	0.1053
Facilities					
Facilities	0.1169	0.1164	0.0604	0.0732	0.0779
Radon					
Radon zone	−0.1703	−0.1877	−0.1909	−0.1606	−0.1753

Complete list of variable loadings is available in Additional file 3.

The loadings for the variables that comprise the sociodemographic domain also varied by RUCC code (Table 9), indicating some variables were more influential in urban settings while others exerted more of an effect on the domain score in rural counties. The patterns of association within the socioeconomic construct were fairly consistent, however, meaning the variables that loaded negatively in the urban counties also loaded negatively in the least urban counties. For instance, renter occupation and vacant units were negatively associated with median household value and median household income across rural–urban status. The one socioeconomic variable for which this was not the case was for the percentage of persons who worked outside the county; for this variable, working outside the county in less urbanized and thinly populated was inversely associated with more than a high school education, but was positively associated in metropolitan urbanized and non-metropolitan urbanized counties. The first principal component accounted for 32% of all county-level variability and was normally distributed.

**Table 9:** Overall and RUCC-stratified loadings for select variables in the sociodemographic domain

**Constructs indented**	**Metropolitan - urbanized**	**Non-metro urbanized**	**Less-urbanized**	**Thinly populated**	**Overall**
	**(RUCC1 = 1089)**	**(RUCC2 = 323)**	**(RUCC 3 = 1059)**	**(RUCC4 = 670)**	**(n = 3141)**
**Sociodemographic domain**					
Socioeconomic					
% renter occupied	0.2344	−0.1665	−0.0246	−0.1235	−0.0374
% vacant units	0.1757	−0.0586	−0.0209	0.0142	−0.1968
Median hh value	−0.1762	0.2484	0.2604	0.2160	0.2907
Median hh income	−0.4096	0.4190	0.4399	0.4545	0.4490
% persons < poverty	0.4535	−0.4568	−0.4728	−0.5169	−0.4557
% no English	0.1562	−0.2656	−0.1923	−0.1847	−0.1252
% earning > high school	−0.3328	0.3673	0.4345	0.4559	0.3925
% unemployed	0.3718	−0.4053	−0.3429	−0.3322	−0.3250
% work outside county	−0.1967	0.1228	−0.0892	−0.0663	0.0996
Median number rooms	−0.4091	0.3314	0.3077	0.2878	0.3501
% > housing 10 units	0.0205	0.1325	0.2289	0.0733	0.2017
Crime					
Log violent crime	0.1728	−0.1039	−0.1251	−0.1385	−0.1325

Complete list of variable loadings is available in Additional file 3.

The variables that comprised the built environment domain loaded much less consistently across the rural–urban categories (Table 10). In general, there were more inverse or negative variable loadings in the most urban counties compared with the less urban counties, and the most rural counties had fairly consistent positive variable loadings. Given this variability, the first principal component accounted for only 23% of the total county-level variability in the built environment, but was also normally distributed.

**Table 10:** Overall and RUCC-stratified loadings for select variables in the built environment domain

**Constructs indented**	**Metropolitan - urbanized**	**Non-metro urbanized**	**Less-urbanized**	**Thinly populated**	**Overall**
	**(RUCC1 = 1089)**	**(RUCC2 = 323)**	**(RUCC 3 = 1059)**	**(RUCC4 = 670)**	**(n = 3141)**
**Built environment domain**					
Roads					
Highway proportion	0.1249	−0.0209	0.1275	−0.0106	0.1320
Primary streets proportion	0.0857	−0.0744	−0.1143	−0.1103	0.0578
Highway/road safety					
Log traffic fatalities	−0.1507	−0.1938	0.0097	0.0272	0.0018
Public transit behavior					
Proportion using public transport	0.2794	0.0635	−0.0212	0.074	0.2058
Business environment					
Log vice-related environment	0.2547	0.2157	0.321	0.3536	0.2687
Log entertainment environment	0.3470	0.4422	0.3822	0.3721	0.3585
Log education environment	0.2405	0.2355	0.2866	0.3713	0.3242
Log negative food environment	0.2147	0.2372	0.2536	0.2514	0.2162
Log positive food environment	0.3666	0.4004	0.4241	0.3127	0.2995
Log health care environment	0.4245	0.4497	0.4653	0.4055	0.4241
Log recreation environment	0.2120	0.3901	0.3309	0.3434	0.2888
Log transportation environment	0.2998	0.1979	0.1985	0.2752	0.3207
Log civic environment	0.2865	0.2114	0.1692	0.2209	0.2912
Subsidized housing environment					
Log total subsidized units	−0.2448	0.0518	0.1440	0.2024	0.2566

Complete list of variable loadings is available in Additional file 3.

### Domain-specific index description for overall EQI

The means, standard deviations, and ranges for each domain-specific index are presented in Table 11. In general, higher values of the air and sociodemographic indices were found in the more metropolitan areas and the most thinly populated areas have the lowest values of each of the indices. Mean values for the land domain index did not vary substantially by RUCC strata and mean values for the built environment indices were below zero, or in the direction of better built environment quality.

**Table 11:** RUCC-stratified description (mean, standard deviation, range) of domain-specific indices used in overall EQI construction for 3141 counties (2000–2005)

**Metropolitan urbanized areas; RUCC 1 (n = 1089)**	**Mean**	**Standard deviation**	**Minimum**	**Maximum**
Air domain index	0.756	0.662	−1.780	2.790
Water domain index	0.052	1.019	−1.641	1.478
Land domain index	0.089	0.909	−5.136	2.095
Sociodemographic domain index	0.594	0.955	−3.027	3.979
Built environment domain index	−0.213	0.878	−4.109	3.884
**Non-metropolitan urbanized areas; RUCC 2 (n = 323)**	**Mean**	**Standard deviation**	**Minimum**	**Maximum**
Air domain index	0.484	0.474	−1.553	1.517
Water domain index	0.111	1.033	−1.570	1.306
Land domain index	0.089	0.909	−5.019	1.479
Sociodemographic domain index	0.023	0.858	−4.810	2.165
Built environment domain index	−0.563	0.485	−1.043	2.165
**Less-urbanized areas; RUCC 3 (n = 1059)**	**Mean**	**Standard deviation**	**Minimum**	**Maximum**
Air domain index	−0.199	0.654	−2.731	1.204
Water domain index	0.066	0.955	−1.565	1.301
Land domain index	−0.069	1.007	−5.139	1.408
Sociodemographic domain index	−0.316	0.854	−4.620	3.127
Built environment domain index	−0.096	0.792	−6.086	3.127
**Thinly populated areas; RUCC 4 (n = 670)**	**Mean**	**Standard deviation**	**Minimum**	**Maximum**
Air domain index	−1.1141	0.879	−3.258	0.7300
Water domain index	−0.241	0.987	−1.555	1.732
Land domain index	−0.072	1.122	−5.210	1.732
Sociodemographic domain index	−0.477	0.860	−4.332	1.263
Built environment domain index	−0.770	1.225	−5.530	2.787

Correlations among the domain specific indices were modest (Table 12), ranging from 0.08 (air and water domain) to 0.40 (air and built domain). The correlations between the overall EQI and each of the domain specific indices reflected the relative importance of that domain to overall environmental variability, and ranged from 0.75 (overall EQI and the sociodemographic domain) to 0.37 (overall EQI and the water domain).

**Table 12:** Pearson correlation coefficients for aim, water, land, sociodemographic, built domains and overall EQI for 3141 U.S. counties (2000–2005)

	**Air domain**	**Water domain**	**Land domain**	**SD domain**	**Built domain**	**Overall EQI**
**Air domain**	1.0					
**Water domain**	0.08	1.0				
**Land domain**	0.09	0.18	1.0			
**SD domain**	0.38	0.11	0.34	1.0		
**Built domain**	0.40	0.16	0.19	0.32	1.0	
**Overall EQI**	0.68	0.37	0.54	0.75	0.71	1.0

### Domain-specific loadings on overall EQI

The first principal component accounted for 39% of the total county-level non-residential ambient environmental variability. The pattern of association for the domain-specific loadings differed by rural–urban status (Table 13). As constructed, the index loadings on the overall EQI index are mean (0) and standard deviation (1); the index is normally distributed with a very slight left skew. In the most urban areas, RUCC 1, the built environment domain was most influential as indicated by its highest loading value (0.52) followed by the air domain (0.51). For the non-metropolitan urbanized areas (RUCC 2), the sociodemographic and land domains loaded similarly on the overall EQI (0.60 and 0.55, respectively), followed by the built environment domain. For this particular grouping of counties, the water domain was least influential, based on its low PCA coefficient (0.30). The air domain was the least influential for the less-urbanized counties ((RUCC 3) 0.16), followed by the water domain (0.30). In the most thinly populated counties, the air and water domain were characterized by the lowest loadings (0.03 and 0.13, respectively) while the sociodemographic and land domains were the most influential (loadings of 0.63 and 0.58, respectively).

**Table 13:** RUCC-stratified domain-specific loadings and 95% confidence intervals (95% CI) on overall EQI for 3141 EQI counties (2000–2005)

**Metropolitan urbanized areas; RUCC 1 (n = 1089**	**Coefficient**	**95% CI**
Air domain index	0.5063	0.4379, 0.5747
Water domain index	0.2757	0.1828, 0.3686
Land domain index	0.4379	0.36552, 0.5107
Sociodemographic domain index	0.4538	0.3945, 0.5131
Built environment domain index	0.5196	0.4565, 0.5827
**Non-metropolitan urbanized areas; RUCC 2 (n = 323)**	**Coefficient**	**95% CI**
Air domain index	0.3343	0.0.80, 0.5705
Water domain index	0.2958	0.0738, 0.5178
Land domain index	0.5506	0.4168, 0.6845
Sociodemographic domain index	0.5963	0.4913, 0.7012
Built environment domain index	0.3769	0.1719, 0.5819
**Less-urbanized areas; RUCC 3 (n = 1059)**	**Coefficient**	**95% CI**
Air domain index	0.1609	0.0477, 0.2740
Water domain index	0.2981	0.1976, 0.3987
Land domain index	0.5503	0.4905, 0.6058
Sociodemographic domain index	0.5675	0.5112, 0.6238
Built environment domain index	0.5102	0.4479, 0.5726
**Thinly populated areas; RUCC 4 (n = 670)**	**Coefficient**	**95% CI**
Air domain index	0.0285	−0.1507, 0.2076
Water domain index	0.1347	−0.0444, 0.3138
Land domain index	0.5785	0.4920, 0.6649
Sociodemographic domain index	0.6263	0.5555, 0.6972
Built environment domain index	0.5041	0.3980, 0.6103

### Description of EQI

**Figure 2:**
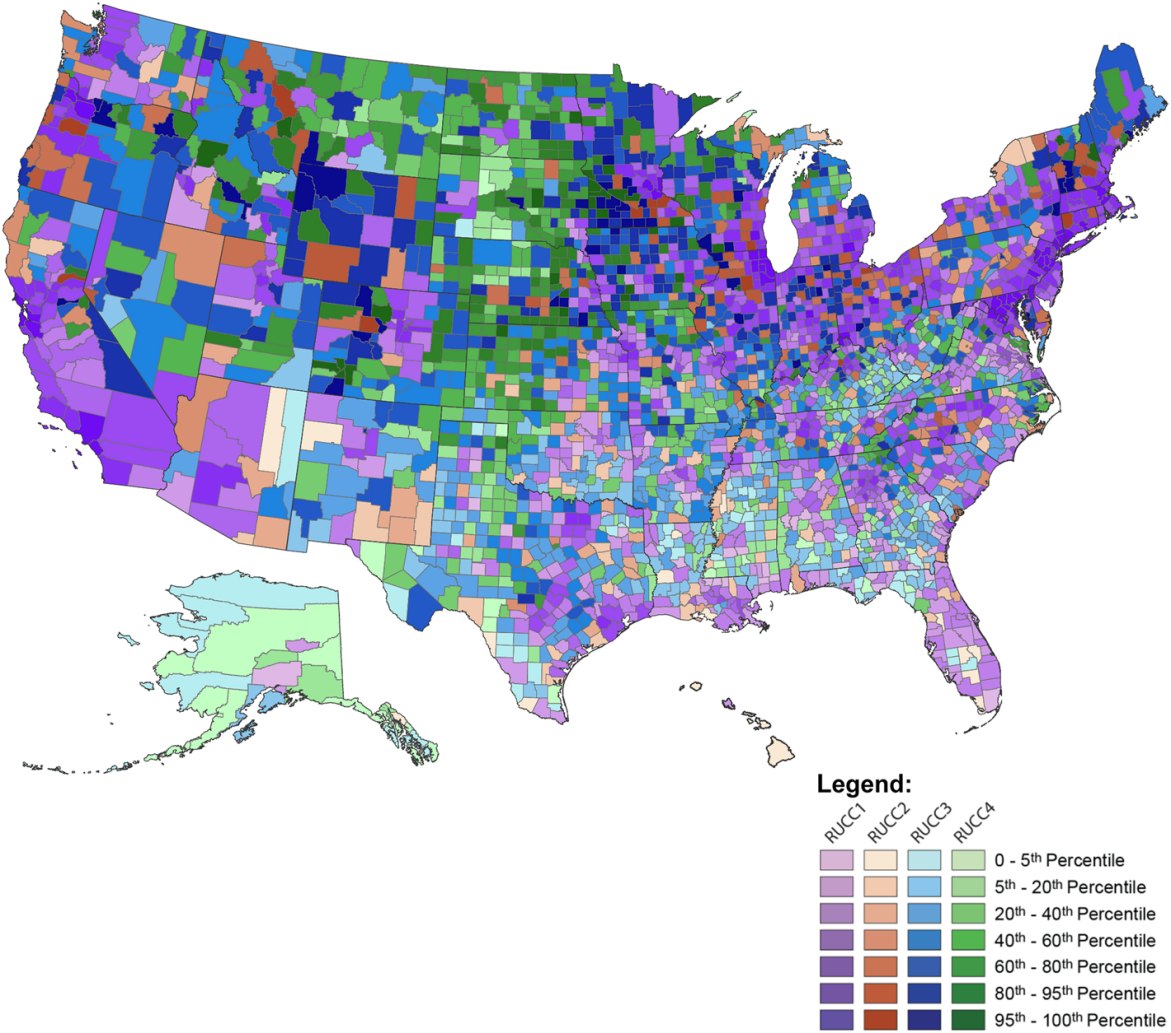
Distribution of overall EQI scores across rural–urban categories for years 2000-2005*.

The distribution of the RUCC-stratified EQI scores is displayed in Figure 2. For these scores, higher values tend toward poorer environments while negative values are associated with more positive domain attributes. By virtue of their standardization, just under half of the EQI score across all RUCC strata were at the negative end of the distribution. The metropolitan urbanized (RUCC 1) and non-metropolitan urbanized (RUCC 2) counties had approximately the same heterogeneity of EQI score (−4.39, 2.53 and −4.74, 2.20, respectively). The less-urbanized counties (RUCC 3) demonstrated the greatest heterogeneity and range of EQI scores (−4.76, 3.57) while the thinly populated strata (RUCC 4) contained counties with the most positive environments (EQI score ranges from −5.86, 2.52).

### Correlations with other sociodemographic features

Environmental quality is only modestly associated with age, sex and racial sociodemographic characteristics in the United States (Table 14). The lowest positive correlations are between the percent under five years of age and high values on the EQI in both the overall and in the most urban counties (0.05 and 0.02, respectively). The highest correlations, 0.60 and 0.54, were for the relationship between percent white non-Hispanic and EQI values in the non-metro and less urban counties.

**Table 14:** Correlations between sociodemographic variables, RUCC-stratified EQI and overall EQI for 3141 U.S. counties (2000–2005)

	**Overall EQI**	**Metro-urban EQI**	**Non-metro EQI**	**Less urban EQI**	**Thin pop / rural EQI**
**Female (%)**	0.199	0.140	0.103	0.141	0.213
**Male (%)**	−0.199	−0.140	−0.103	−0.141	−0.213
**Under 5 (%)**	0.047	0.024	−0.316	−0.304	−0.211
**Over 65 (%)**	−0.209	−0.173	0.106	0.222	0.238
**White non-Hispanic (%)**	0.262	0.191	0.600	0.535	0.422
**Black non-Hispanic (%)**	−0.167	−0.267	−0.412	−0.385	−0.189

## Discussion

We developed an Environmental Quality Index for all counties in the United States incorporating data for five environmental domains: air, water, land, built, and sociodemographic. For each environmental domain, variables were constructed to represent exposures within that domain; indices for each domain and for environmental quality as a whole were developed by stratifying by rural–urban continuum codes. Variable loadings varied by domain and rural–urban designation, suggesting that environmental quality is driven by different domains in rural and urban areas. By virtue of the standardization used to construct the indices, approximately equal numbers of counties were at the positive end of the environmental quality spectrum as were at the negative end of the environmental quality spectrum.

The EQI is not the only index available for environmental estimation. The Environmental Performance Index (EPI), produced by a team at Yale University, is a country-level index that uses 22 performance indicators for which countries can be held accountable for environmental sustainability [53]. Both the EQI and the EPI rely on similar data sources (official statistics, monitoring data, modeled data, spatial data), prepare data similarly for variable construction (e.g., use of population denominators to construct standardized weights), and employ weighting and aggregation in construction. These similarities support the approach undertaken to construct the EQI. The EPI differs from the EQI in important ways, however. The EPI includes a substantially different set of environmental domains than the EQI, focusing on water effects (human and ecological health), air effects (human and ecological health), biodiversity and habitat, forests, fisheries, agriculture, climate change and energy. It is also constructed using target-based indicators for assessing performance on environmental health indicators rather than being purely an environmental representation. Finally, the EPI is aggregated at the country level to accommodate its international scope, while the EQI, though solely for the United States, gets at much finer detail at the county level.

Another index for natural environment vulnerability was developed by the South Pacific Applied Geoscience commission, the United Nations Environment Programme and their partners. The Environmental Vulnerability Index (EVI) [54] was developed through collaboration with countries, institutions, and experts across the globe and was designed for use with other economic and social vulnerability indices to provide insights into the processes that can negatively influence the sustainable development of countries. The EVI is based on 50 indicators for estimating country-level environmental vulnerability. Unlike the EQI, it is constructed by averaging the various measures. One limitation of the EVI is that it does not reflect environments dominated by human systems (e.g., cities, farms).

Most other environmental quality indices focus on one environmental domain (e.g., Air Quality Index [55]) or a specific type of activity (e.g., Pedestrian Environmental Quality Index [56]) or vulnerability (e.g., Cumulative Environmental Vulnerability Assessment [57], heat vulnerability index [58]). State-specific indices also exist, (e.g., CalEnviro Screen 1.0 [59], Virginia Environmental Quality Index [60] and Michigan Environmental Quality Index [61]) but their comparability across states is limited by their respective data sources and construction. A major strength of the EQI is that it encompasses multiple environmental domains, and all U.S. states and counties.

The EQI holds substantial promise for improving environmental estimation for public health. One important limitation of prior environmental health work has been the inability to control for the multiple environments to which people are simultaneously exposed. If these multiple human activity spaces occur within the same county, using the EQI will provide an estimate of the non-residential ambient county-level conditions to which residents are exposed, whether they are at home, at school, or at work. In addition to the EQI, each of the domain-specific indices is informative. The domain-specific loadings on the EQI indicate which of the environmental domains accounts for the largest portion of the variability in the EQI; in essence, these loadings answer the question as to which domain is making the biggest contribution to the total environment. Because most environmental health practice occurs at the domain level, this domain-specific information may be even more important to policy makers and environmental health activists than the overall EQI. Drilling down further, the variable loadings on each of the domains are also informative for the same reason. In the land environment, for instance, it might be important to know if pesticides or superfund sites seem to be contributing the largest share of variability to the land index. This information has obvious implications for public health intervention. The RUCC-stratified domains and EQI indices will also make an important public health contribution. We know urban and rural areas differ in important ways and these RUCC-stratified indices help us disentangle what domains may be driving some of the observed rural–urban differences in public health outcomes. While the total amount of environmental variability accounted for by any given EQI domain or the overall index may be modest, they contribute more control for or explanation of non-residential environmental conditions than has heretofore been possible.

While the process and product reported here makes a clear contribution to the environmental health literature, this work is not without limitations. Despite the large number of variables used for the EQI, data scarcity – in terms of spatial and temporal coverage – represents an important limitation to this work. Many of the data sources required spatial or temporal kriging to construct county level estimates. For example, even with extensive air monitoring networks, the measured spatial coverage of the U.S. is incomplete, particularly in rural areas. Many data sources are disproportionately located in urban areas (e.g., crime data), whereas others are found in rural areas (e.g., industrial livestock operations). The nonrandom distribution of environmental risk means that virtually all interpolated data are inaccurate, and our ability to draw inference for data-sparse rural areas is impaired.

Another potential limitation of the EQI is its construction at the county level. While the county may be too diffuse a unit to enable specific exposure assessment, it is a fair representation of the non-residential ambient environment. By explicitly describing the EQI construction process, we provide the necessary tools for interested investigators to apply at smaller units of aggregation with more specific data sources. Further, we plan to provide access to the data used to construct the EQI publically on the U.S. EPA website. A third limitation results from the data that were available for EQI construction. One aspect of our literature review identifying data sources used “infant mortality and environment” as search terms. While we contend we obtained adequate representation of the five environmental domains, it is possible our use of infant mortality precluded us from finding an environmental domain. Despite this possibility, however, the index is so broadly representative of the non-residential ambient environment it should be widely applicable to other health outcomes. Most of the EQI data were collected for non-research purposes; therefore, the data collection methodology, quality control and reporting varied across data source, domain and variable. We endeavored to include comparable data whenever possible, but data-quality differences are important to recognize. Because we relied on available data, and not all sources of environmental quality are measured at the county level, not all potentially relevant data are represented in the EQI. However, we attempted to capture as much as available for each of the five domains. Further, more data are collected in urban areas, which likely results in a more valid estimate of urban compared with rural environments. We have little information for Native American reservations and National Parks, for instance, which limits our ability to comment on those county spaces. In addition, the use of the EQI as a measure of exposure assumes exposure to “environment” is consistent for all individuals, but the extent of environmental exposure was not assessed. The EQI is focused mostly on the outside environment, which may not be the most relevant exposure in relation to human health and disease. Finally, population-level analyses offer little predictive utility for individual-level risk. Therefore, while the index may be useful at identifying lower quality environments that may predict population-level health outcomes, it cannot be used to predict adverse outcomes for individuals. We believe the EQI, and the approach taken for its development, represents a promising step and we encourage others to contribute additional work to this endeavor.

## Conclusions

The Environmental Quality Index was constructed for all counties in the United States and incorporates a wide variety of data to provide a broad picture of environmental conditions in the United States. The approach we undertook was based on a reproducible methodology that accesses mostly publically-available data sources. Future development of the EQI includes assessing the consequences of the variable choices through sensitivity analyses, updating for 2006–2010, and exploring other levels of spatial aggregation. In this manuscript we present a valid, easily replicable methodology that can be broadly applied at different units of aggregation. As environmental public health researchers, we are fundamentally interested in the environmental contribution to human health. The EQI may aid us in developing knowledge on connections between the overall environment and human health outcomes.

## Abbreviations

AQS: Air quality system; EPI: Environmental performance index; EQI: Environmental quality index; EPA: U. S. environmental protection agency; EVI: Environmental vulnerability index; FARS: Fatality annual reporting system; FBI: Federal bureau of investigation; FIPS: Federal information processing standards; HAPs: Hazardous air pollutants; HUD: Housing urban development; NATA: National-scale air toxics assessment; NCHS: National center for health statistics; NCOD: National contaminant occurrence database; NDAP: National atmospheric deposition program; NGS: National geochemical survey; NPDES: National pollutant discharge elimination system; NPL: National priority list; PCA: Principal components analysis; PM2.5: Particulate matter under 2.5 micrometers in aerodynamic diameter; PM10: Particulate matter under 10 micrometers in aerodynamic diameter; ROE: Report on the environment; RUCC: Rural urban continuum code; U.S.: United States; TIGER: Topologically integrated geographic encoding and referencing; USGS: United states geological survey; WATERS: Watershed assessment tracking < environmental results.

## Competing interests

The authors declare that they have no competing interests.

## Authors’ contributions

LCM contributed to the study conception, constructed the EQI and drafted the manuscript. JSJ contributed to the EQI construction and manuscript revisions. KMR contributed to the EQI construction and manuscript revisions. DTL conceived of the study, participated in its design and coordination, and manuscript revisions. All authors read and approved the final manuscript.

## Supplementary Material

Additional file 1 Methods Appendix. Click here for file

Additional file 2 Overall and rural-urban continuum codes (RUCC)-stratified domain variable means, standard deviations and ranges. Click here for file

Additional file 3 Overall and rural-urban continuum codes (RUCC)-stratified loading for all variables. Click here for file
