# Supplementary material for: Construction of an environmental quality index for public health research
Source: Environ Health. 2014 May 22;13:39. doi: 10.1186/1476-069X-13-39 (PMC4046025; doi:10.1186/1476-069X-13-39)
Supplement: Additional file 1 — Methods Appendix. [file 1476-069X-13-39-S1.pdf]

Additional file 1 – methods appendix

Domain identification.
We initially identified three environmental domains, air, water and land, based on selected chapters from the U.S. Environmental Protection Agency (EPA) 2008 Report on the Environment (ROE) [1]. Following consultation with the ROE, the team undertook a more extensive review to complement the domains and data sources already identified, which included the following activities: 1) Identified precise literature search terms, limits and reporting format; 2) conducted a literature review on “Environment and Infant Mortality”; 3) recorded findings; 4) finalized search terms for within-domain literature review; 5) conducted within domain literature review; 6) recorded findings. We chose infant mortality to be the health outcome for the literature search for several reasons: 1) infant mortality is a well-researched and understood health outcome; 2) infant mortality is a general outcome, with known positive associations with other lifetime-health measures like disability-adjusted life expectancy [2], and the environmental exposure – health outcome relationship would not be restricted to one organ (e.g., heart disease) or system (e.g., asthma); 3) the research team was largely composed of reproductive / perinatal researchers for whom infant mortality was an important health outcome. The literature review was conducted in PubMed, which is a service of the U.S. National Library of Medicine and the National Institutes of Health for the years 1980-2008. We added the built and social demographic domains based on the findings of the literature review. This search process revealed specific indicators that have been used to estimate the association of sub-domains and infant mortality. For instance, after searching under “air environment and infant mortality”, one would find literature assessing carbon monoxide (CO), particulate matter, ozone, etc. We then used each of these indicators as key terms to use in subsequent searches. The team also explored references from the main papers, defined as those that come up repeatedly in the various searches, to make sure seminal papers, indicators, sub-domains or domains were not missed. From this broad search, and our a priori identification, five specific domains were considered: air, water, land, built, and sociodemographic environments.

Geographic level of analysis.
The unit of analysis for EQI development was U.S. county. While county is a broad unit of analysis that may not allow for small-geography specificity, most national data sources are available at the county-level. We wanted to construct a replicable process and product for use across the United States and we deemed the county-level as the most widely generalizable. It also allows for linking to those health data aggregated to the county level, such as national birth statistics from the National Center for Health Statistics (NCHS).

Data source time period.
At the initiation of the EQI development, we restricted the temporal framework to 2000 to 2005. We wanted to primarily utilize publicly available data, and this six-year window was chosen based on availability of both environmental (including decennial census) and outcome data (e.g., national birth records).

Data sources.
The data sources are described in detail elsewhere[3]. Briefly, data sources were considered for EQI inclusion based on temporal, spatial, and quality-related criteria. Temporal appropriateness required data to represent the 2000 to 2005 time period. Data sources were considered spatially appropriate if data were available at, or could be

aggregated or kriged to represent, the county-level for all 50 states. Kriging is a geospatial technique that uses known data points to interpolate data at locations with unknown measurements[4]. Data quality, especially related to data source documentation, was determined by data source managers (in data reports and internal documentation), project investigators, and with the larger field of environmental research, through use and critique of the various data sources.

The air domain included two data sources: the Air Quality System (AQS)[5], which is a repository of national ambient air concentrations from monitors across the country for criteria air pollutants; and the National-Scale Air Toxics Assessment (NATA)[6], which uses emissions inventory data and air dispersion models to estimate non-residential ambient concentrations of hazardous air pollutants (HAPs).

The water domain comprised five data sources: Watershed Assessment, Tracking & Environmental Results (WATERS) Program Database [7], Estimates of Water Use in the U.S. [8], National Atmospheric Deposition Program (NDAP) [9], Drought Monitor Network[10], and National Contaminant Occurrence Database (NCOD) [11]. The WATERS Program Database is a collection of data from various EPA-conducted water assessment programs including impairment, water quality standards, pollutant discharge permits, and beach violations and closures. The Estimates of Water Use in the U.S. is calculated by the United States Geological Survey (USGS) and includes county level estimates of water withdrawals for domestic, agricultural, and industrial uses. The NDAP dataset provides measures of chemicals in precipitation using a network of monitors located throughout the U.S. The Drought Monitor Data provides raster data on the drought status for the entire U.S. on a weekly basis. The NCOD dataset provides data from public water supplies on 69 different contaminants.

The land domain was constructed using data from five sources. The 2002 National Pesticide Use Database [12] estimates state-level pesticide usage based on pesticide ingredients and crop type. The 2002 Census of Agriculture [13] is a summary of agricultural activity, including information about crops, livestock, and chemicals used. The National Priority Site data [14] includes location of and information on sites that have been placed on the National Priority List (NPL), including indicators for major facilities (e.g., Superfund sites), large quantity generators, toxics release inventory, resources conservation and recovery act treatment, storage and disposal facilities (and corrective action facilities, assessment, cleanup, and redevelopment exchange (brownfield sites), and section seven tracking system pesticide producing site locations. The National Geochemical Survey [15] contains geochemical data (e.g., arsenic, selenium, mercury, lead, zinc, magnesium, manganese, iron, etc). The fifth source is the EPA Radon Zone Map [16], which identifies areas of the U.S. with the potential for elevated indoor radon levels.

The sociodemographic domain included two data sources: the U.S. Census [17]and Federal Bureau of Investigation (FBI) Uniform Crime Report (UCR) [18]. The U.S. Census collects population and housing data every 10 years, economic and government data every five years and the American Community Survey annually. FBI UCR rate data are available annually and by crime type (violent or property).

The built environment domain employed five data sources. Dun and Bradstreet collects commercial information on businesses and contains more than 195 million records [19]. These data are the only publically available data, which are not available for free, used

in the EQI. Topographically Integrated Geocoding Encoding Reference (TIGER) [20] data provides maps and road layers for the U.S. at multiple units of census geography. The Fatality Analysis Reporting System (FARS) [21] data is a national census providing the National Highway Traffic Safety administration yearly reports of fatal injuries suffered in motor vehicle crashes. Housing and Urban Development (HUD) [22] data provide a count of low-rent and section-eight housing in each housing authority area, which correspond to cities. The built environment domain also included one census variable; census data have been previously described.

EQI construction.

Variable construction. Each of the data sources could plausibly give rise to hundreds of potentially relevant variables; therefore only specific variables were selected – or in some cases constructed – from each of the data sources. Some variables have been widely used and were therefore obvious choices. For example, air quality is frequently estimated using measures of particulate matter or ozone, while the sociodemographic conditions frequently include some measure of poverty[23-25]. Other variables, particularly those in the water domain, needed to be constructed from multiple existing data sources. A detailed listing of all the constructed variables is available in **Additional file 2**.

Statistical processes common to all variables in all domains. Variable collinearity was assessed within subgrouping and when the correlation coefficients exceeded 0.7, one variable was chosen for inclusion. Variables with low numbers of missing values were retained over those with high numbers of missing values. If missingness was approximately equal, the decision about which variable to retain was based on exposure routes from hazard summaries [26], with routes from the appropriate domain being primary.

Variable missingness was also assessed to determine if missing data were missing or instead represented true zeros. For instance, when crime data was missing for a county we considered that missing but when low-income housing data were missing for a county, we considered those to be true zeros. When more than 50 percent of all counties were missing or zero for a given variable, that variable was excluded from further consideration for the EQI.

Because of the data reduction approach used for index construction (principal components analysis (PCA), discussed in detail below), and the statistical assumptions implied by this method, variables were assessed for normality. This was done by visually comparing histograms of each variable's distribution to a normal distribution for that variable. When violations of normality were visually observed, transformations were considered to enable the variable to best approximate the normal distribution. For each variable, natural-log, logit, and squared-root transformations were considered and distributions were visually inspected again. In each case, log transformation resulted in the most normally-appearing distribution. For variables with true zeros, log-transformation was achieved by adding half of the non-zero minimum value to all observations then taking the natural log of that value.

Finally, variables were assessed to determine valences for environmental quality. Valences, or the positive or negative direction of the indices, were determined based on potential for human health and ecological effects. Domains containing variables with

known or suspected potential for adverse health outcomes (e.g., increased morbidity or mortality) or ecologic effects (e.g., disruption of biotic integrity) were considered to have a negative valence with higher values representing poorer environmental quality. In some cases, the valence of a given variable was unknown, in which case the valence would be empirically assigned through the data reduction / PCA process by virtue of its association with other variables in that domain. The specifics of variable construction for each of the domains are presented below.

Air domain variable construction: Daily concentrations of six criteria air pollutants were downloaded from the AQS[5] and temporally averaged to get annual mean concentrations for each monitor location from 2000 to 2005. The annual means were then temporally and spatially kriged to estimate annual concentrations at each county center point. An exponential covariance structure for the spatial covariance was implemented to represent both temporal and spatial variability. These values were then averaged for the full study period.

The 2002 NATA [6] database was used as an initial source of county-level HAP concentrations for evaluation of variable inclusion. After evaluation for collinearity (18 variables removed) and missingness (77 variables removed), 81 HAPs were considered appropriate for EQI inclusion; emissions estimates for these were retrieved from the NATAs for 1999 (40 available) and 2005 (81 available), and estimates for each variable from the three NATAs were averaged to get a composite emissions estimate across the study period. Air domain variables were then checked for normality of distribution and where indicated were log-transformed (85 of 87 variables; $PM_{2.5}$ and carbon tetrachloride were not transformed). For both criteria and hazardous air pollutants, higher concentrations are negative for air quality. Therefore, the valence of the air domain is negative.

Water domain variable construction: Water impairment is determined for multiple types of water usage: agricultural, drinking, recreational, wildlife and industrial. Using the WATERS [7] database and joining the data in GIS software with measures of stream length in the Reach Address Database [27], we developed 11 variables for water impairment [28]. However, only one was used due to county-level missingness. A cumulative measure of percent of water impaired for any use was used to represent overall water quality in the county. A high percent of impaired waters represents poor water quality and therefore the valence for this variable is negative.

Water contamination can be caused by several sources and we used the number of National Pollutant Discharge Elimination System (NPDES) [29] permits in a county as a proxy for general water contamination. Using permit information in the WATERS database, we calculated 13 variables for the number of discharge permits in a county. Because the 10 variables which were calculated based on individual permit types had too many missing values, the three composite variables were included in the EQI. We developed a composite variable for number of sewage permits per 1000 km of stream length in a county by summing the number of Animal Feeding Operations/Concentrated Animal Feeding Operations NPDES permits, Combined Sewer Overflow NPDES permits, and NPDES permits for sludge in each county and dividing by the total stream length in the county. Similarly, we calculated composite variables for industrial permits (combining total of pretreatment NPDES permits, general facilities NPDES permits, and individual facilities NPDES permits) and stormwater permits (combining total of general stormwater NPDES Permits, industrial stormwater NPDES permits) county per 1000 km

of stream length. These three variables were not collinear. A high number of pollution permits is considered poor for water environmental quality and therefore the valence for these variables is negative.

Recreational water quality was assessed also using the WATERS database [7] which also includes annual information on the number of days of beach closures, from which we created three variables for the number of days of beach closure for any event in a county, the number of days of beach closure for contamination events in a county, and the number of days of beach closure for rain events in a county. Overall, the seven variables constructed from these data were not collinear. A high percent of impaired waters, high number of pollution permits, and a high number of beach closures are associated with poorer water quality - and therefore the valence for these variables is negative.

The quality of the water used for domestic needs data was extracted from the Estimates of Water Use in the U.S. [8] database as a proxy for domestic water quality. Initially 15 variables of water withdrawals for domestic, agricultural, and industrial use were developed. After evaluation for collinearity (four variables removed) and missingness (nine variables removed), two variables were included in the EQI: the percent of population on self-supplied water supplies and the percent of those on public water supplies which are on surface waters. These variables are not associated with good or bad water quality and therefore the valence is neutral for this component of the water domain.

The atmospheric deposition of chemicals can affect water quality. The NDAP [9] dataset provides measures for the concentration of nine chemicals in precipitation, calcuim, magnesium, potassium, sodium, ammonium, nitrate, chloride, sulfate, and mercury. Annual summary data from each monitoring site for each year 2000-2005 were spatially kriged, using an exponential covariance structure, to achieve national coverage and county level estimates. The annual estimates for each pollutant were then averaged over the six-year study period. The data for all pollutants, except sulfate, were skewed and therefore were log- transformed to achieve normal distributions. No variables were removed for collinearity or missingness. Higher concentrations of these chemicals are considered harmful to water quality, therefore the valence for this component of the water domain is negative, in the direction of poor environmental quality.

We expected that drought affects the concentration of pathogens and chemicals in waters and therefore can affect water quality. The Drought Monitor [10] dataset provides raster data on six possible drought status conditions for the entire U.S. on a weekly basis. The data were spatially aggregated to the county level to estimate the percentage of the county in each drought status condition. From this data we used the percentage of the county in extreme drought (D3-D4) in the EQI. The remaining five drought status conditions were removed as all of the drought statuses were highly correlated. Drought can have negative impacts on water quality and therefore the valence is negative for this component of the water domain.

Chemical contamination of water supplies was extracted from the NCOD [11] dataset which provides data on 69 contaminants provided by public water supplies throughout the country for the period from 1998-2005. Data for all samples in a county for each contaminant were averaged over the entire time period of the data. The data were also log-transformed to achieve normal distributions. Missing values were set to zero, with

the assumption that lack of measurement for an area indicated low concern for contamination with that particular contaminant. Eight contaminants, asbestos, diquat, endothall, glyphosate, dioxin, radium, beta particles, and uranium, were not represented in enough counties (missingness) to be included in the EQI. No variables were deleted for collinearity. Higher concentrations of these contaminants is considered harmful to water quality; therefore, the valence for this component of the water domain is negative.

The majority of variables in the water domain are estimates of pollutants for which higher values are considered negative for water quality. The final valence of the water domain is negative, indicating a higher water domain score is associated with poorer environmental quality.

Land domain variable construction: Information on the agricultural environment, including non-pesticide chemicals used in farming, harvested acreage, irrigated acreage, and proportion of farms were obtained from the 2002 Census of Agriculture [13]. Agricultural animal units were estimated by multiplying the number of livestock by the "animals per animal unit" statistic, then summing across livestock categories [30]. In total, eight variables representing agriculture were constructed and county-level percentages (acres applied per county total acreage) were calculated. Following normality assessment, all agriculture variables were log-transformed. While the presence of agriculture is not in and of itself negative, these variables represent the presence of non-naturally occurring chemicals and the potential for environmentally disruptive practices. Therefore, the valence of the agricultural construct was considered negative.

Variables specific to pesticide application were also constructed. Herbicide, insecticide, and fungicide use for each county were estimated using crop data from the 2002 Census of Agriculture and state pesticide use data from the 2002 National Pesticide Use Dataset [12]. County level acreage for specific crops was multiplied by state level pesticide use rates (tons/acre) to estimate pesticide use. All pesticide variables were log-transformed. In general, exposure to pesticides is not generally considered positive for human or ecologic health; therefore, the valence for the pesticide components of the land domain was considered negative, which equates with poor environmental quality.

The natural geochemistry and soil contamination of an area was estimated using the National Geochemical Survey (NGS) data [15]. These data, collected for stream sediments, soils, and other media, were combined at the county level to estimate the mean values of 13 geochemical contaminants available. Contaminant variables were evaluated for normality and all were log-transformed. While many of these contaminants are naturally-occurring, excess concentrations can be harmful to human and ecologic health; therefore the valence for soil contaminants is negative and higher values are representative of poor environmental quality.

Large industrial facilities represent sources for pollutants released into the environment. The National Priority List [14] data from the EPA provided information on facilities for the U.S.. Because many counties had at least one, but no counties had all six of the facility types present, a composite facilities data variable was constructed by summing the count of any one of the six facilities types (brownfield sites (n=1226) [31], superfund sites (n=721) [32], toxic release inventory sites (n=2670) [33], pesticide producing location sites (n=2095) [34], large quantity generator sites (n=1926) [35], and treatment, storage and disposal sites (n=874) [36]) across the counties. The composite count of facilities

was divided by the county population, which produced a facilities rate. The facilities rate variable was assessed for normality and log-transformed. Because the presence of these facilities has the potential to be harmful to human and ecologic health, the valence of facilities per capita is negative, with higher values representing poor environmental quality.

Finally, the potential for elevated indoor radon levels was represented using county score from the EPA Radon Zone map [16]. While radon is naturally-occurring, high levels are harmful to human health. Therefore the valence for higher amounts of this portion of the land domain represents poor environmental quality.

As all constructs in the land domain were determined to have a negative valence, the valence of the land domain as a whole is also negative, indicating a higher land domain score represents poor environmental quality.

Sociodemographic domain: The sociodemographic environment is an important environment for human health. Eleven variables from the United States Census [17] were included in the sociodemographic domain of the EQI. The 11 variables were percent renter-occupied housing, percent vacant housing units, median household value, percent persons living below the federal poverty line, percent no English-speaking, percent earning more than a high school education, percent unemployed, percent working outside the county of residence, median number of rooms in the housing unit, and percent of housing with more than 10 units. Following normality assessment, the percent of housing with more than 10 units was log-transformed before inclusion in the EQI. The sociodemographic domain contains a mix of positive and negative features; therefore when the sociodemographic domain was constructed, positive variables were reverse-coded to ensure that a higher amount of the sociodemographic domain represented adverse environmental conditions.
The area-level crime environment was represented using the Federal Bureau of Investigation (FBI) Uniform Crime Reports (UCR) [18]. These data required some manipulation for inclusion in the EQI. First, each jurisdiction or place, the unit at which crime data is reported, was assigned to a county Federal Information Processing Standards (FIPS) code. In cases when a jurisdiction covered more than one county, the reported crime was assigned to both counties. While this double assignment results in a slight inflation of crime reports for a state, there was no way to determine which county should receive the crime reports. Further, if police or municipal jurisdictions crossed county lines, it is likely residents of both counties were "exposed" to the crime environment. This crime data being attributed to more than one county occurred in approximately 15 counties. Second, because crime reporting is voluntary and crime data are reported for less than half the U.S. counties, yet it seemed unlikely that no crimes occurred in the areas with no reported crime, crime data were spatially and temporally kriged to estimate values for counties with no reported crime. Kriging employed a double exponential covariance structure for the spatial covariance; one structure represented short-range variability and the other long-range variability. The covariance model was fit to experimental covariance values using a least squares method and demonstrated sufficient fit. Varying geographical unit sizes were not explicitly accounted for through the kriging estimates, but crime estimates were made for 57 percent of U.S. counties, mostly in rural areas. The crime variable was log-transformed for inclusion in the EQI. Living in areas with high crime rates is detrimental to human health and well-being, therefore the valence of these data is negative.

Both constructs in the sociodemographic domain have a negative valence. Therefore, the final valence of the sociodemographic domain is negative, indicating a higher sociodemographic domain score is associated with poor environmental quality.

Built environment domain: Housing environments vary and features of the housing environment have the potential to influence human health and well-being. The housing environment was represented using two variables available from the HUD data source, low-rent and section-eight [37]. These variables were summed to result in the count of any low-rent or section-eight housing in each county. The rate of subsidized housing was constructed by dividing the count of subsidized housing units per county by the county population. Normality of the subsidized housing rate was assessed and this variable was log-transformed. The presence of public housing has typically been a marker for poverty and poor housing conditions, which have been associated with poor health. Therefore the valence for the public housing variables is toward poor environmental quality.

Highway safety was represented by a traffic fatality variable. Rates for the count of fatal crashes per county were constructed by dividing the count of county-level fatal crashes (FARS) [21] by the county-level population. This rate was log distributed (due to many counties having zero fatal crashes) and was therefore log-transformed before inclusion. Traffic fatalities are a marker for hazardous road conditions, which have the potential for negative impacts on human health. The valence for traffic fatality is negative, toward poor environmental quality.

The percent of county residents who use public transportation was the only U.S. Census [17] variable used in the built environment domain of the EQI. While this variable is available for all 3141 counties, for many counties, the percent of the population who reports using public transportation is near 0. Therefore, this variable was log-transformed prior to its use in the built domain of the EQI. Public transportation use may convey multiple meanings therefore the valence for this variable is neutral.

We were interested in characterizing the relative proportions of each county that were served by highways, secondary roads and primary roads. Two proportion variables were constructed from the TIGER data [20] by dividing the mileage of each road type (e.g., secondary primary roads) by the total road mileage in each county. The proportions of all roadways that were highways or primary roads, available for 3141 counties, were included. Similar to the public transportation use variables, proportions of roadways of varying types is varying in its likely association with human health and ecologic impact; therefore the valence of this variable’s construct for roadways is neutral for environmental health.

Business and service environments are important predictors of human health and activity. We sought to estimate features of the economic and service environment using data from Duns and Bradstreet [19]. Nine business environment rate variables were constructed by dividing the county-level count of a business type by the county-level population count. The nine variables that were constructed from the Dun and Bradstreet data include: the positive food environment, negative food environment, vice environment (alcohol, pawn, gaming), entertainment environment, health care business environment, recreation environment, education environment, social-service environment and transportation-related environments. All variables except the negative food environment were log-transformed for normality. The business and service

environments contain a mix of positive and negative features; therefore when the built domain was constructed, positive variables were reverse-coded to ensure that a higher amount of the these service variables represent adverse environmental conditions. The built domain's valence is negative and indicating a higher built domain score represents poor environmental quality.

EQI temporal representation. Variable consistency (mean and standard deviation) was compared across each year of the six-year period (2000-2005). Additionally, proto-EQIs were constructed using data from one year (2002) and from the average of all six-years. For those variables that were spatially kriged, county-level values before and after kriging were also compared. Because these county-level values were temporally consistent, the EQI was constructed based on county-level averages for the six-year period for each variable in each domain.

RUCC stratification. Recognizing that environments differ across the rural – urban continuum [38], we concluded the EQI would be most useful if it accommodated rural-urban environmental differences. Therefore, EQI construction was stratified by rural-urban continuum codes (RUCC). The RUCC is a nine-item categorization code of proximity to/influence of major metropolitan areas [39]. As has been done elsewhere, the nine-item categories were condensed into four categories for which RUCC1 represents metropolitan urbanized = codes 1+2+3; RUCC2 non-metro urbanized = 4+5; RUCC3 less urbanized = 6+7; and RUCC4 thinly populated =8+9 [40-43]. Both stratified county-specific and all-county indices were created. Loadings on the stratified and non-stratified sets of indices were assessed to determine loading heterogeneity across counties. Because these loadings differed meaningfully by RUCC level, we constructed a RUCC-stratified EQI for each county.

Data reduction. Similar to the approach employed in other research [23, 44, 45], principal components analysis (PCA) was chosen for data reduction in this study because the investigators sought an empirical summary of total area-level variance explained by the environmental variables, rather than a confirmation of any underlying factor structure comprised of the previously identified domains.

Component extraction and index construction. The constructed variables from each dataset were merged to produce a domain-specific county-level dataset. The domain-specific variables were then combined using PCA. PCA produces variable loadings, which are roughly equivalent to the "weight" or contribution that each variable makes toward explaining the total variance. The loading associated with each variable is then multiplied by its mean value for the given geography (county, for the EQI) and these weighted mean values are summed. Although it is possible to form as many independent linear combinations as there are variables, we retained only the first principal component: the unique linear combination that accounted for the largest possible proportion of the total variability in the component measures. This process was undertaken separately for each of the four RUCC strata.

Frequently in index construction, variables with low loadings may be excluded to produce a more parsimonious index. Both within and across each RUCC strata, domain-specific variable loadings were evaluated based on the variable's hypothesized relevance to health. For instance, while mercury may be a chemical with notable concentrations only in limited areas across the U.S. and may therefore have a small component loading, it is an important health hazard when present. So based on variable

loading magnitude alone, one might consider dropping mercury from an environmental quality index but we instead decided to retain it based on its relevance to human health. Because it was unclear which of the variables included in the domain-specific PCAs were irrelevant to human health, we retained all the variables for inclusion in the RUCC-stratified and overall indices.

The first principal component, which we labeled the domain-specific index (e.g., air domain index), was standardized to have a mean of 0 and standard deviation (SD) of 1 by dividing the index by the square of the eigenvalue [46]. Each domain-specific index was then included in a second PCA procedure (**Figure 1**), from which we extracted the first principal component to create the overall EQI for each strata of RUCC.

References

1. United States Environmental Protection Agency (EPA): **EPA's 2008 Report on the Environment**. In. Washington, DC; 2008.
2. Reidpath DD, Allotey P: **Infant mortality rate as an indicator of population health**. *J Epidemiol Community Health* 2003, **57**:344-346.
3. Lobdell DT, Jagai JS, Rappazzo K, Messer LC: **Data sources for an environmental quality index: availability, quality, and utility**. *Am J Public Health* 2011, **101 Suppl 1**:S277-285.
4. Lee SJ, Serre ML, van Donkelaar A, Martin RV, Burnett RT, Jerrett M: **Comparison of geostatistical interpolation and remote sensing techniques for estimating long-term exposure to ambient PM2.5 concentrations across the continental United States**. *Environ Health Perspect* 2012, **120**:1727-1732.
5. **The Ambient Air Monitoring Program** [http://www.epa.gov/air/oaqps/qa/monprog.html]
6. **National Air Toxics Assessments** [http://www.epa.gov/ttn/atw/natamain/]
7. United States Environmental Protection Agency (EPA): **Watershed Assessment, Tracking and Environmental Results (WATERS)**. In.; 2010.
8. **Estimated Use of Water in the United States** [http://water.usgs.gov/watuse/]
9. **National Atmospheric Deposition Program** [http://nadp.sws.uiuc.edu/]
10. **U.S. Drought Monitor** [http://droughtmonitor.unl.edu/monitor.html]
11. **National Contaminant Occurrence Database (NCOD)** [http://water.epa.gov/scitech/datait/databases/drink/ncod/databases-index.cfm]
12. Gianessi L, Reigner N: **Pesticide Use in U.S. Crop Production: 2002. Insecticides & Other Pesticides**. In. Washington, D.C.: CropLife Foundation; 2006.
13. **2002 Census of Agriculture full report** [http://www.agcensus.usda.gov/Publications/2002/index.asp]
14. **EPA's web feature service for National Priority List (NPL) sites** [http://geodata.gov]
15. **National geochemical survey** [http://tin.er.usgs.gov/geochem/doc/averages/countydata.htm]
16. **Map of radon zones** [http://www.epa.gov/radon/zonemap.html]
17. [(http://factfinder.census.gov)]
18. **Uniform Crime Reports** [http://www.fbi.gov/ucr/ucr.htm]
19. **Dun and Bradstreet Products** [http://www.dnb.com/us/dbproducts/product_overview/index.html]
20. **Topologically Integrated Geographic Encoding and Referencing** [http://www.census.gov/geo/www/tiger/]

21. **Fatality Analysis Reporting System (FARS)** [http://www.nhtsa.gov/people/ncsa/fars.html]
22. **HA Profiles List** [https://pic.hud.gov/pic/haprofiles/haprofilelist.asp]
23. Messer LC, Laraia BA, Kaufman JS, Eyster J, Holzman C, Culhane J, Elo I, Burke JG, O'Campo P: **The development of a standardized neighborhood deprivation index**. *J Urban Health* 2006, **83**:1041-1062.
24. El-Sayed AM, Scarborough P, Galea S: **Socioeconomic inequalities in childhood obesity in the United Kingdom: a systematic review of the literature**. *Obesity facts* 2012, **5**:671-692.
25. Reiss F: **Socioeconomic inequalities and mental health problems in children and adolescents: a systematic review**. *Soc Sci Med* 2013, **90**:24-31.
26. **Health Effects Notebook for Hazardous Air Pollutants** [http://www.epa.gov/ttn/atw/hlthef/hapindex.html]
27. **Reach Address Database** [http://www.epa.gov/waters/doc/rad/index.html]
28. Jagai JS, Rosenbaum BJ, Pierson SM, Messer LC, Rappazzo K, Naumova EN, Lobdell DT: **Putting regulatory data to work at the service of public health: Utilizing data collected under the Clean Water Act**. *Water Quality, Exposure, and Health* 2013, **5**:117-125.
29. **National Pollutant Discharge Elimination System (NPDES)** [http://cfpub.epa.gov/npdes/home.cfm?program_id=45]
30. Kellog R.L. LCH, Moffitt D.C., Gollehon N.: **Manure Nutrients Relative to the Capacity of Cropland and Pastureland to Assimilate Nutrients: Spatial and Temporal Trends for the United States**. In.: United States Department of Agriculture; 2000.
31. **Assessment, Cleanup, and Redevelopment Exchange (ACRES) Brownfield Sites** [http://www.epa.gov/brownfields/]
32. **Superfund National Priorities List (NPL) Sites** [http://www.epa.gov/superfund/sites/npl/index.htm]
33. **Toxics Release Inventory (TRI) Sites** [http://www.epa.gov/tri/]
34. **Section Seven Tracking System (SSTS) Pesticide Producing Site Locations** [http://www.epa.gov/compliance/data/systems/toxics/sstsys.html]
35. **Resource Conservation and Recovery Act (RCRA) Large Quantity Generators (LQG)** [http://www.epa.gov/osw/hazard/generation/lqg.htm]
36. **Resource Conservation and Recovery Act (RCRA) Treatment, Storage, and Disposal Facilities (TSD) and (RCRA) Corrective Action Facilities** [http://www.epa.gov/osw/hazard/tsd/index.htm]
37. **Multifamily Assistance and Section 8 Contracts Database** [http://portal.hud.gov/hudportal/HUD?src=/program_offices/housing/mfh/exp/mfhdiscl]
38. Hall SA, Kaufman JS, Ricketts TC: **Defining urban and rural areas in U.S. epidemiologic studies**. *J Urban Health* 2006, **83**:162-175.
39. **Measuring rurality: Rural-urban continuum codes.** [http://www.ers.usda.gov/Briefing/Rurality/ruralurbcon/.]
40. Langlois PH, Jandle L, Scheuerle A, Horel SA, Carozza SE: **Occurrence of conotruncal heart birth defects in Texas: a comparison of urban/rural classifications**. *J Rural Health* 2010, **26**:164-174.
41. Messer LC, Luben TJ, Mendola P, Carozza SE, Horel SA, Langlois PH: **Urban-rural residence and the occurrence of cleft lip and cleft palate in Texas, 1999-2003**. *Ann Epidemiol* 2010, **20**:32-39.

42. Langlois PH, Scheuerle A, Horel SA, Carozza SE: **Urban versus rural residence and occurrence of septal heart defects in Texas**. *Birth Defects Res A Clin Mol Teratol* 2009, **85**:764-772.
43. Luben TJ, Messer LC, Mendola P, Carozza SE, Horel SA, Langlois PH: **Urban-rural residence and the occurrence of neural tube defects in Texas, 1999-2003**. *Health Place* 2009, **15**:848-854.
44. Genberg BL, Gange SJ, Go VF, Celentano DD, Kirk GD, Latkin CA, Mehta SH: **The effect of neighborhood deprivation and residential relocation on long-term injection cessation among injection drug users (IDUs) in Baltimore, Maryland**. *Addiction* 2011, **106**:1966-1974.
45. Doubeni CA, Schootman M, Major JM, Stone RA, Laiyemo AO, Park Y, Lian M, Messer L, Graubard BI, Sinha R, Hollenbeck AR, Schatzkin A: **Health status, neighborhood socioeconomic context, and premature mortality in the United States: The National Institutes of Health-AARP Diet and Health Study**. *Am J Public Health* 2012, **102**:680-688.
46. Kim J-O. MCW: **Factor Analysis: Statistical Methods and Practical Issues**, vol. 07-014. Newbury Park, CA: Sage; 1978.
